# Accuracy of the Apple Watch Series 4 and Fitbit Versa for Assessing Energy Expenditure and Heart Rate of Wheelchair Users During Treadmill Wheelchair Propulsion: Cross-sectional Study

**DOI:** 10.2196/52312

**Published:** 2024-05-07

**Authors:** Marius Lyng Danielsson, Melanie Vergeer, Guy Plasqui, Julia Kathrin Baumgart

**Affiliations:** 1 Centre for Elite Sports Research Department of Neuromedicine and Movement Science Norwegian University of Science and Technology Trondheim Norway; 2 Department of Nutrition and Movement Sciences Maastricht University Maastricht Netherlands

**Keywords:** agreement, validity, accuracy, cross sectional, physiology, disability, disabled, upper-body exercise, upper body, exercise, physical activity, ergospirometer, fitness, vital, vitals, energy, expenditure, mHealth, wearable, wearables, mobile health, smartwatch, smartwatches, apple watch, fitbit, digital health, energy expenditure, heart rate, wheelchair, wheelchairs, fitness trackers, tracker, trackers

## Abstract

**Background:**

The Apple Watch (AW) Series 1 provides energy expenditure (EE) for wheelchair users but was found to be inaccurate with an error of approximately 30%, and the corresponding error for heart rate (HR) provided by the Fitbit Charge 2 was approximately 10% to 20%. Improved accuracy of estimated EE and HR is expected with newer editions of these smart watches (SWs).

**Objective:**

This study aims to assess the accuracy of the AW Series 4 (wheelchair-specific setting) and the Fitbit Versa (treadmill running mode) for estimating EE and HR during wheelchair propulsion at different intensities.

**Methods:**

Data from 20 manual wheelchair users (male: n=11, female: n=9; body mass: mean 75, SD 19 kg) and 20 people without a disability (male: n=11, female: n=9; body mass: mean 75, SD 11 kg) were included. Three 4-minute wheelchair propulsion stages at increasing speed were performed on 3 separate test days (0.5%, 2.5%, or 5% incline), while EE and HR were collected by criterion devices and the AW or Fitbit. The mean absolute percentage error (MAPE) was used to indicate the absolute agreement between the criterion device and SWs for EE and HR. Additionally, linear mixed model analyses assessed the effect of exercise intensity, sex, and group on the SW error. Interclass correlation coefficients were used to assess relative agreement between criterion devices and SWs.

**Results:**

The AW underestimated EE with MAPEs of 29.2% (SD 22%) in wheelchair users and 30% (SD 12%) in people without a disability. The Fitbit overestimated EE with MAPEs of 73.9% (SD 7%) in wheelchair users and 44.7% (SD 38%) in people without a disability. Both SWs underestimated HR. The device error for EE and HR increased with intensity for both SWs (all comparisons: *P*<.001), and the only significant difference between groups was found for HR in the AW (–5.27 beats/min for wheelchair users; *P*=.02). There was a significant effect of sex on the estimation error in EE, with worse accuracy for the AW (–0.69 kcal/min; *P*<.001) and better accuracy for the Fitbit (–2.08 kcal/min; *P*<.001) in female participants. For HR, sex differences were found only for the AW, with a smaller error in female participants (5.23 beats/min; *P*=.02). Interclass correlation coefficients showed poor to moderate relative agreement for both SWs apart from 2 stage-incline combinations (AW: 0.12-0.57 for EE and 0.11-0.86 for HR; Fitbit: 0.06-0.85 for EE and 0.03-0.29 for HR).

**Conclusions:**

Neither the AW nor Fitbit were sufficiently accurate for estimating EE or HR during wheelchair propulsion. The AW underestimated EE and the Fitbit overestimated EE, and both SWs underestimated HR. Caution is hence required when using SWs as a tool for training intensity regulation and energy balance or imbalance in wheelchair users.

## Introduction

Wheelchair users are generally less active than people without a disability, which increases their risk of developing noncommunicable diseases such as cardiovascular disease, type 2 diabetes, and obesity [1-4]. In fact, the prevalence of obesity is 2.5 times greater in wheelchair users compared to people without a disability [5]. This is related to an energy intake that exceeds their energy expenditure (EE), which is approximately 5% to 40% lower in wheelchair users compared to people without a disability [6-8].

Total EE in people without a disability is approximately 1900 to 2900 kcal/day and comprised of 3 components: 60% to 75% attributed to resting EE (REE), 10% to diet-induced thermogenesis, and 15% to 30% to physical activity EE (PAEE) [9,10]. The lower total EE of wheelchair users is mainly related to reduced REE and PAEE [11-13]. Wheelchair users with a spinal cord injury (SCI) have a 14% to 27% lower REE due to reduced fat-free mass and sympathetic nervous system activity [11]. Furthermore, wheelchair users have lower PAEE due to a smaller amount of active muscle mass during upper-body exercise compared to what ambulatory people without a disability expend during walking or running [14,15]. Also, PAEE typically ranges between 6% and 36% of the total daily EE in wheelchair users with a SCI [16,17] and is the most modifiable of the 3 components. Therefore, PAEE may be particularly useful for obtaining a balance between EE and energy intake.

Criterion devices for measuring EE, such as direct or indirect calorimetry, are restricted to the laboratory setting and expensive to use. Therefore, more accessible devices that accurately estimate EE within the population of wheelchair users are needed. Smart watches (SWs) are widely used to provide feedback on estimated EE and monitor physical activity intensity (eg, through monitoring heart rate [HR]) [14,18-20]. If sufficiently accurate, the feedback provided by SWs may serve as a tool to counteract obesity and promote physical activity in wheelchair users.

Commonly used cutoffs for acceptable accuracy of parameters provided by wearable devices are ±10% in free-living settings and ±3% in standardized settings [18,21]. However, even in standardized settings, SWs often estimate HR and EE values outside of this range in both wheelchair users and people without a disability [18-22]. Additionally, the development of wheelchair user–specific estimation algorithms for EE and HR is especially challenging because of the high heterogeneity and disability-related differences in physiological functioning in this population. Currently, the only study that evaluated the accuracy of estimated HR with a commercially available SW (Fitbit Charge 2 [Fitbit Inc]) found lower accuracy for wheelchair users (mean absolute percentage errors [MAPE] of approximately 10%-20% dependent on level of SCI) compared to people without a disability (approximately 8% MAPE) [23]. Furthermore, the sole study that assessed the accuracy of the estimated EE in the commercially available Apple Watch (AW) Series 1 (Apple Inc) with a wheelchair-specific setting reported a MAPE of 29% [24]. Notably, both studies report that the measurement error for estimating HR and EE increased with higher-intensity exercise [23,24]. While follow-up studies have not yet been conducted, one would expect companies to further improve the accuracy of their HR and EE estimation algorithms.

Therefore, the aim of this study was to assess the accuracy of the AW Series 4 (in the wheelchair-specific setting “outdoor push walking pace”) and the Fitbit Versa (in the treadmill running mode) for estimating EE and HR during wheelchair propulsion at different intensities. We decided to include both wheelchair users and a control group consisting of people without a disability to investigate if the wheelchair setting was specifically adjusted for wheelchair users.

## Methods

### Participants

A total of 20 wheelchair users and 20 people without a disability were included in the study. Both groups consisted of 11 male participants and 9 female participants and had similar demographic characteristics (Table 1). Participants were included if they were aged between 18 and 60 years and without injury or other health issues that could be aggravated by physical exertion. Included in the wheelchair user group were individuals that used a manual wheelchair as a main form of transport or were ambulatory wheelchair users. The wheelchair user group was comprised of individuals with SCI (n=11), spina bifida (n=2), and cerebral palsy (n=2). A total of 5 participants had other neurological, musculoskeletal, or joint impairments. Participants were recruited from sports associations and organizations for people with disabilities in Norway and social media.

**Table 1:** Participant characteristics.

Groups and sex			Age (years), mean (SD)		Body mass (kg), mean (SD)		Body height (cm), mean (SD)		BMI (kg/m^2^), mean (SD)
**Combined**									
	All	35.3 (11.8)		74.8 (15.2)		174.5 (10.9)		24.5 (4.1)	
	Male participants	36.3 (12.2)		81.1 (11.9)		181.9 (7.2)		24.4 (2.8)	
	Female participants	34.1 (11.5)		67.1 (15.6)		165.3 (7.1)		24.6 (5.3)	
**Wheelchair users**									
	All	37.4 (12.6)		74.5 (18.6)		172.5 (12.2)		24.9 (5.3)	
	Male participants	40.0 (12.9)		80.4 (14.3)		180.5 (8.5)		24.5 (3.3)	
	Female participants	34.1 (12.1)		67.2 (21.3)		162.7 (8.0)		25.3 (7.2)	
**People without a disability**									
	All	33.3 (10.8)		75.2 (11.4)		176.2 (9.9)		24.2 (2.4)	
	Male participants	32.6 (10.8)		81.9 (9.4)		183.5 (5.9)		24.3 (2.4)	
	Female participants	34.0 (11.5)		67.0 (7.8)		167.3 (5.3)		23.9 (2.6)	

### Study Protocol

Three test days of wheelchair propulsion with different treadmill incline-speed combinations were conducted within 2 consecutive weeks. A minimum of 24 hours separated each test day, and sessions occurred at approximately the same time of day to account for diurnal variations. All test days started with a 5-minute warmup at a 0.5% incline at a self-chosen speed that corresponded to a rating of perceived exertion of 7-9 on the Borg scale [25]. Then, 3 standardized 4-minute stages were performed at a predetermined incline for the day (either 0.5%, 2.5%, or 5%) with increasing speed across the stages. The order of the test days was counterbalanced. The speed at each incline was established through pilot testing and determined to be manageable for the participants (Table 2). Anthropometric data (age and sex) were collected before testing, and body mass and height were collected on the first test day. Participants were instructed to avoid high intensity training and alcohol consumption 24 hours before testing, avoid caffeine on the day of testing, and fast for at least 2 hours before testing.

**Table 2:** Overview of the standardized speeds for the 3 test days (0.5%, 2.5%, or 5% incline) for male participants (without tetraplegia) and female participants or male tetraplegic wheelchair users.

Test days and participants		Stage 1, speed (km/h)		Stage 2, speed (km/h)		Stage 3, speed (km/h)	
**0.5% day**							
	Male participants		4		6		8
	Female participants or male participant with tetraplegia		3		5		7
**2.5% day**							
	Male participants		3		4		5
	Female participants or male participant with tetraplegia		2		3		4
**5% day**							
	Male participants		2		3		4
	Female participants or male participant with tetraplegia		1		2		3

### Equipment

Participants’ body mass was measured using a Kistler force plate (Kistler 9286BA; Kistler Instruments AG) before the first test day. Body mass was determined for participants in the wheelchair user group while seated in their own wheelchair and obtained by subtracting the mass of the individual wheelchair (range 6.5-18.2 kg). All people without a disability were weighed while standing without any equipment. Participants wore a facemask (7450 V2 Series; Hans Rudolph Inc), which was connected to a Vyntus CPX ergospirometer with a mixing chamber (Vyaire, Medical GmbH) to measure gas exchange as 10-second averages, from which the criterion device EE was calculated. The Vyntus CPX was calibrated against a known gas mixture of 15% O_2_ and 5% CO_2_ before every test. Participants were fitted with a Polar HR monitor (version M400; Polar Electro Oy) and a Polar chest strap (version H10; Polar Electro Oy), which served as the criterion device for HR.

**Figure 1:**
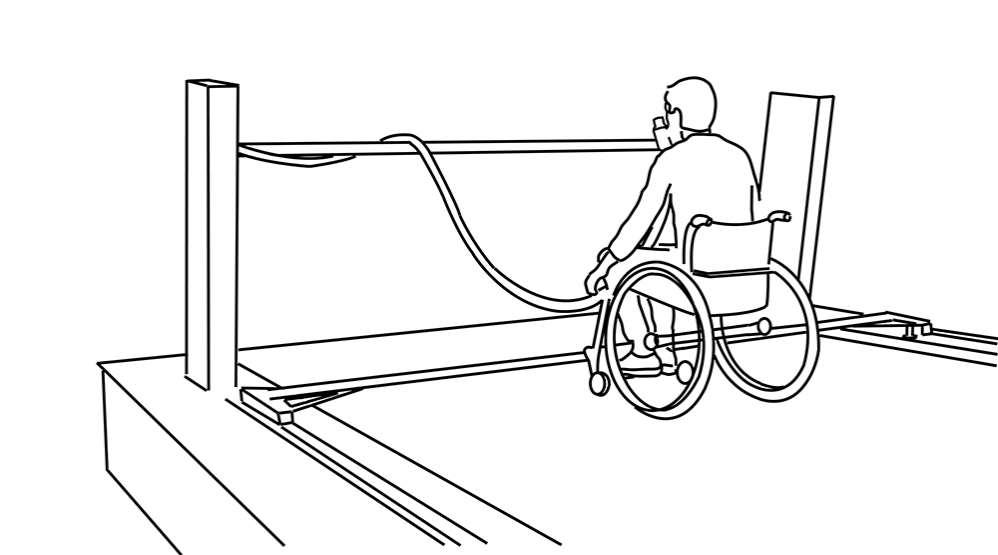
Test setup on the treadmill with the manual wheelchair attached to the traverse safety bar.

Participants wore 2 SWs on their nondominant wrist: an AW Series 4 software version OS 7.3.3 and a Fitbit Versa (2017) software version OS 5.0. The SWs tracked HR using photoplethysmography, and both SWs had a built-in accelerometer and gyroscope. The SW placement (closest to the wrist) was counterbalanced. Participant characteristics were entered in the devices, and the activity settings of “outdoor push walking pace” (AW) and “treadmill running” (Fitbit) were used throughout testing. “Treadmill running” was chosen for the Fitbit in the absence of a wheelchair-specific setting. wheelchair users used their personal wheelchair, and people without a disability used a standardized wheelchair (Küschall K-Series Attract; Invacare; mass 11.7kg). The wheelchairs were secured on a motorized 5×3 m treadmill (Forcelink Technology) with a mobile traverse bar attached to side rails (Figure 1). The side rails were equipped with safety stoppers to prevent participants from rolling off the back of the treadmill.

Blood lactate concentrations (mmol/L) were measured in a rested state and after every stage from a 20-μl blood sample obtained from the participants earlobe [26]. Concentrations were analyzed using the Biosen C-Line Sport lactate measurement system (EKF Industrial Electronics). Rating of perceived exertion was obtained for muscular, respiratory, and total effort on the 6-20 Borg scale after each stage [25].

### Data Analysis

The criterion EE was calculated for every 10-second average VO_2_ and VCO_2_ values using the Weir formula [27]:

EE (kcal/min) = 3.941 × VO_2_ (L/min) + 1.106 × VCO_2_ (L/min)

An average over the entire 4-minute period (as opposed to a steady-state average) was calculated for EE and HR for each stage of the criterion device. This was done since the SWs displayed an estimated average EE and HR for each entire stage. It was not possible to retrieve data with a higher time resolution from the SWs. The EE values provided by the criterion device and SWs were converted to kcal/min for comparison. Lastly, incomplete 4-minute stages were excluded from the analyses.

### Missing Data

In total, 20 of the 360 performed stages were incomplete and excluded from the analyses. Incomplete stages mostly occurred at the highest speeds and often at the 5% incline, which was due to a combination of an age- or disability-related lack of physical fitness or upper-body strength. Additionally, equipment failure or human error contributed to the following missing data: incorrect activity setting (AW, n=2), no HR recorded after activity (AW, n=32), and a lost HR signal (criterion, n=1).

### Statistical Analysis

Statistical analyses were conducted, and visualizations created in R Studio (version 4.2.1; R Core Team) [28]. Descriptive statistics were calculated for EE and HR for criterion devices, AW, and Fitbit and visualized with box plots using the R Studio package *ggplot2* (Multimedia Appendix 1).

#### Absolute Agreement

The MAPE was used to establish the difference between criterion devices and SWs for both EE and HR during each stage:

MAPE = 1/n x ∑[( |C_p_-SW_p_| ) / |C_p_| ] x 100

where criterion devices are represented by *C*, smart watches as *SW*, participants as *p*, and the total number of participants that completed the respective stage as *n*. In addition to the separate MAPEs visualized in a figure, an overall MAPE is provided in text, with the mean and SD being calculated across all participants and stages. The MAPE was categorized based on commonly used accuracy cutoffs for measuring EE, HR, and steps with wearable devices, with an acceptable error of ±10% in free living settings and ±3% in standardized settings [18,21]. Our categorization for the MAPE was therefore as follows: poor (>20%), moderate (10.1%-20%), good (3.1%-10%) and excellent (0%-3%).

The results of linear mixed model analyses are presented in our main results, in addition to Bland-Altman plots in the Multimedia Appendix 2. The main reason for choosing linear mixed model analyses as our main analyses was the repeated-measures design of our data collection with corresponding dependency in data [29], since all participants conducted several stages. The linear mixed model analyses were used to assess if there was a significant difference in EE and HR between criterion devices and SWs and to investigate the effect of group and sex as well as the increase in intensity on these device differences. Speed and incline were not adjusted for in the mixed model analyses, as we were interested in the estimation error of the SWs across all intensities and not within each speed-incline combination. As such, we also did not need to adjust for multiple comparisons. Participant ID was included as a random-intercept effect in these models to account for dependency in the data. An α value of .05 was used to indicate statistical significance. There was no deviation of the residuals from normality (checked by visual inspection of Q-Q plots) and no violation of the assumption of homoscedasticity (checked by plotting the fitted values against the residuals; *plot_model* function, R *sjPlot* package). The inclusion or exclusion of outliers did not change the results of the mixed model analyses. We therefore decided to include the analyses with outliers, as they represent the actual estimation errors of the AW.

#### Relative Agreement

Interclass correlation coefficients (ICCs) were used to quantify relative agreement between criterion devices and SWs for both EE and HR. The ICCs were calculated using the 2-way random effects model with absolute agreement by using the *icc* function from the *irr* package. ICCs were categorized based on widely used cutoff points into poor (<0.5), moderate (0.5-0.75), good (0.75-0.9), and excellent relative agreement (>0.9) [30,31].

### Ethical Considerations

The data collection and processing were approved by the Norwegian Centre for Research Data (216680) and conducted in accordance with the Declaration of Helsinki. All participants were informed of the study purpose, design, potential risks, and the possibility to withdraw without penalty before signing the consent form. Participation was voluntary and without financial compensation beyond insight into the individual’s collected data. The collected data were deidentified.

## Results

### Absolute Agreement

**Figure 2:**
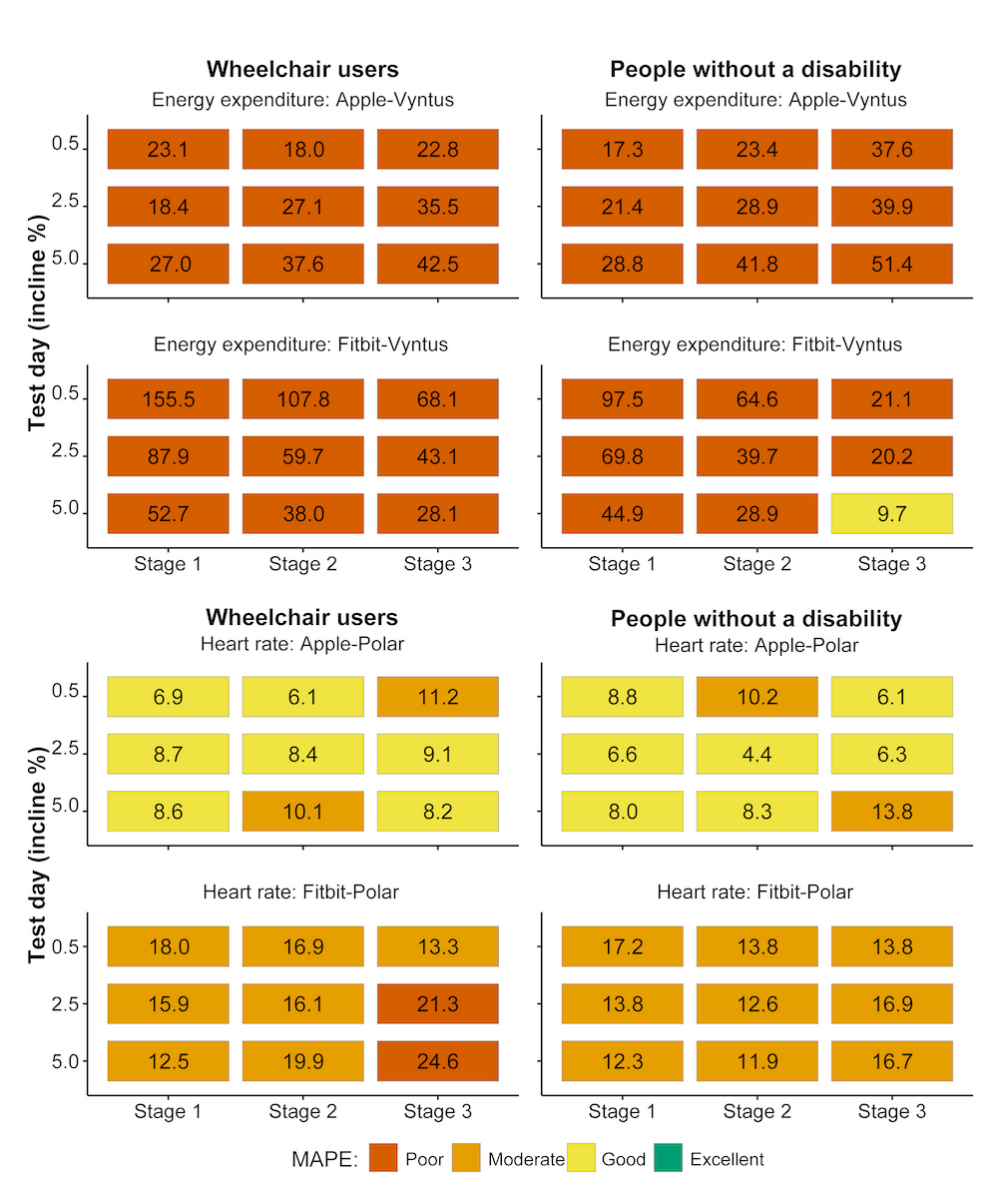
Mean absolute percentage error (MAPE) between criterion devices and smart watches for energy expenditure and heart rate on all 3 test days (0.5%, 2.5%, and 5% incline) and stages with increasing speed. Values are presented separately for wheelchair users and people without a disability. MAPEs were categorized as poor (>20%), moderate (10.1%-20%), good (3.1%-10%), and excellent (0%-3%).

For the EE reported by the AW Series 4, the MAPE (with all inclines and stages combined) was 27.4% (SD 16.7%) in wheelchair users and 32.1% (SD 14.4%) in people without a disability. The EE provided by the Fitbit Versa had a MAPE of 73.9% (SD 57.2%) in wheelchair users and 44.7% (SD 37.8%) in people without a disability. Absolute agreement based on the MAPE for each incline-stage combination was mostly poor (Figure 2).

For HR, the MAPE with all stages and inclines combined was 8.5 (SD 10.4%) in wheelchair users and 8.1 (SD 13.6%) in people without a disability for the AW, and 17.4 (SD 12.4%) in wheelchair users and 14.3 (SD 10.7%) in people without a disability for the Fitbit. The absolute agreement for HR in each incline-stage combination was moderate-good for the AW and poor-moderate for the Fitbit (Figure 2).

**Figure 3:**
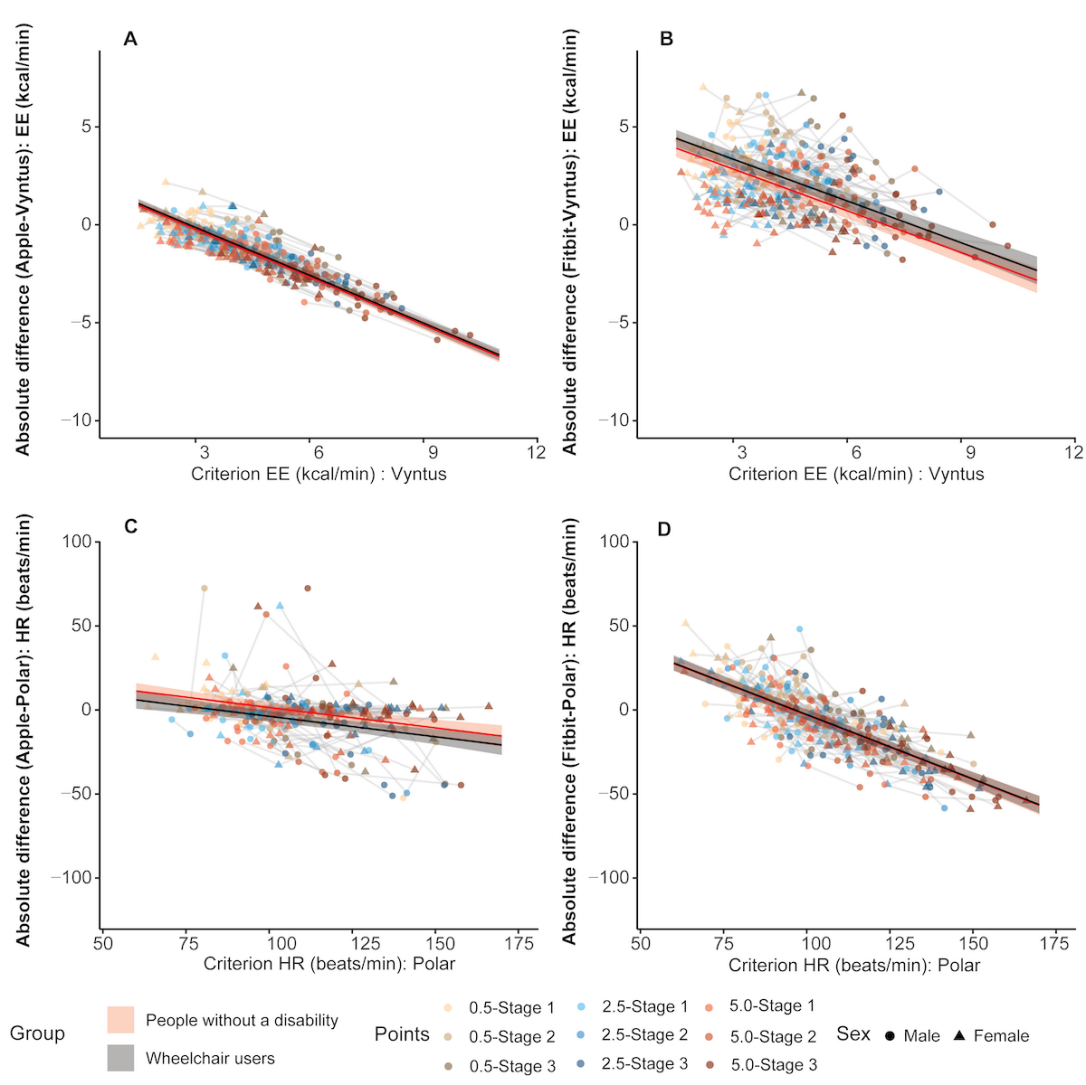
Regression lines (with shaded 95% CIs) separated by groups for the differences in energy expenditure (EE) and heart rate (HR) between smart watches (SWs) and criterion devices based on linear mixed model analyses: (A) EE: Apple-Vyntus; (B) EE: Fitbit-Vyntus; (C) HR: Apple-Polar; and (D): HR: Fitbit-Polar. The x-axis shows the criterion device values, while the y-axis shows the absolute difference between SWs and criterion devices. A regression line below zero indicates underestimation of SWs compared to criterion devices, while 1 above zero indicates overestimation.

The mixed model analyses indicated that EE was underestimated by the AW and overestimated by the Fitbit, while both SWs underestimated HR (Figure 3). Additionally, the differences between criterion and comparison devices increased negatively with higher exercise intensity for EE and HR (all comparisons, *P*<.001; Figure 3 and Multimedia Appendix 2). This led to reduced accuracy in the AW (larger underestimation) and improved accuracy in the Fitbit (lower overestimation) with increased intensity. EE and HR differences between SWs and criterion devices were not significantly different in wheelchair users compared to people without a disability in most comparisons (*P*>.06), with the exception of the HR reported by the AW (–5.27 beats/min; *P*=.02). Furthermore, for EE, the differences were significantly larger in female participants for the AW (–0.69 kcal/min; *P*<.001) and in male participants for the Fitbit (–2.08 kcal/min; *P*<.001). For HR, the only sex difference was found for the AW, with smaller differences between the AW and criterion device in female participants (5.23 beats/min; *P*=.02).

### Relative Agreement

**Figure 4:**
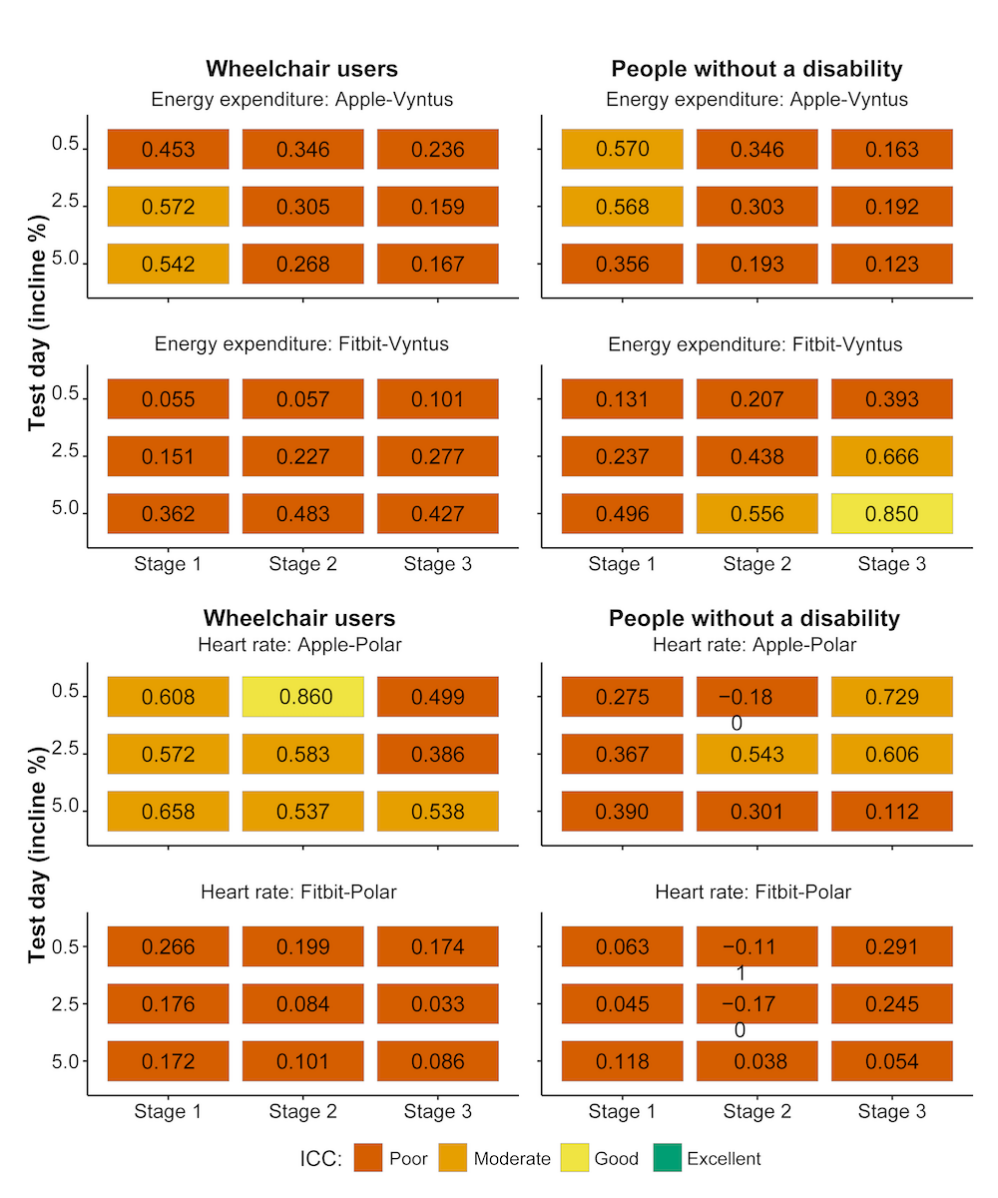
Interclass correlation coefficients (ICCs) between criterion devices and smart watches (SWs) for wheelchair users and people without a disability on all 3 test days (0.5%, 2.5%, and 5% incline) and stages with increasing speed. ICCs were categorized as poor (<0.5), moderate (0.5-0.75), good (0.76-0.9), and excellent (>0.9) for energy expenditure and heart rate.

Apart from 1 EE (Figure 4; a 5% incline for stage 3) and 1 HR ICC (Figure 4; a 0.5% incline for stage 2), all remaining ICCs indicate poor to moderate relative agreement between the criterion devices and SWs (Figure 4). The ICCs for each incline-stage combination for the AW had a range from 0.12 to 0.57 for EE and from 0.11 to 0.86 for HR. For the Fitbit, the corresponding ranges were from 0.06 to 0.85 for EE and from 0.03 to 0.29 for HR.

## Discussion

### Summary

The aim of this study was to assess the accuracy of the AW Series 4 and the Fitbit Versa for estimating EE and HR during wheelchair propulsion at different intensities. The AW underestimated EE and the Fitbit overestimated EE, suggesting that neither of the SWs are accurate enough for estimating EE in wheelchair users. Furthermore, both the AW and Fitbit underestimated HR. Lastly, the differences in HR and EE between SWs and criterion devices increased with increasing intensity, and they mostly did not differ between groups.

### EE Findings

The MAPE of approximately 30% and poor to moderate ICCs of the AW Series 4 are similar to the ones reported by Moreno et al [24] for the AW Series 1. Since the participant characteristics were similar those in this study, there seems to be no improvement in the AW’s EE estimation algorithms. The SW algorithms are proprietary technology, and it cannot therefore be determined why the AW underestimates EE for all participants. A plausible explanation is that Apple intentionally chose to report lower values for the sake of obesity prevention. Another possible explanation is that the data for developing the AW algorithms were collected from wheelchair users with lower training status or higher levels of SCI, who have a lower EE compared to the wheelchair users tested in this study. If the latter is the case, injury and fitness levels are important factors to consider when estimating the EE of wheelchair propulsion.

In this study, the AW was found to be equally inaccurate when estimating EE for wheelchair users and people without a disability, a finding that also aligns with Moreno et al [24]. Since the AW does not request user information on, for example, impairment levels, this finding indicates that the watch’s software is not capable of identifying these types of individual characteristics from other factors such as movement patterns or HR responses. In contrast, sex could be inputted into the AW settings, and we found a significantly better estimated EE for male participants. Preliminary evidence [32] indicates that algorithms for wearable devices are developed mostly based on reference data from male participants and may therefore be less accurate for female participants.

The AW’s underestimation of EE increased with intensity, which contradicts findings from previous AW studies [24,33]. While Pope et al [33] reported lower accuracy at moderate compared to low and high intensity during running, Moreno et al [24] reported consistent accuracy across wheelchair propulsion stages with an increasing stroke rate. The test protocol with the standardized stroke rate increases in Moreno et al [24] might more closely resemble the way the AW estimation algorithm works. Possibly, the AW uses the accelerometry data to determine how much exercise intensity, and thereby EE, has increased. In contrast, in this study, intensity increased with higher speeds at a given incline, with the steeper incline days being more physiologically taxing with a larger anaerobic contribution than the flatter incline days. Furthermore, it is possible that the AW EE estimation algorithms were developed mostly based on low-intensity data or without a physiological intensity measure (eg, HR) since wheelchair users spend most of their day at or below low-intensity exercise levels. In line with this, preliminary findings of our research group [34] indicate that estimation algorithms developed for wheelchair users perform less well on high-intensity data if they are only developed based on low- to moderate-intensity data.

The Fitbit did, in contrast to the AW and other SW studies on EE [24,33], show a systematic decrease in the error with higher intensities for both groups, which resulted in lower overestimations. This finding was most likely related to using the “treadmill running mode” in the absence of a wheelchair-specific setting. This setting leads the estimation algorithm to expect a weight-bearing and leg-dependent activity with higher muscle activation. However, wheelchair propulsion is a non–weight-bearing activity that allows longer rest between cycles, especially at lower speeds and inclines. The reduction of the Fitbit error at higher intensities might therefore be a result of more active muscle mass, faster cycle rates, and longer cycle lengths with increased incline or speed. As such, the physiological effort of wheelchair propulsion may be more similar to running at higher intensities. Although the Fitbit displayed improved accuracy at higher intensities, the MAPEs were far greater than the ±3% acceptable accuracy cutoff, and the Fitbit should therefore not be used to estimate EE in wheelchair users.

### HR Findings

Both SWs showed better accuracy for HR compared to EE. However, only the AW had a MAPE below the arbitrary cutoff of ±10% that is commonly used for acceptable accuracy in free-living activities, with no values below the ±3% cutoff for standardized settings [18,21]. Both SWs additionally showed reduced accuracy at the highest intensities, which is in agreement with previous findings of HR measured from wrist-worn devices during running [22]. Furthermore, wheelchair users were found to have a larger underestimation of HR (ie, –5 beats/min) compared to people without a disability. While the reasons for this are somewhat unclear, it seems like this is due to more negative outliers in wheelchair users. Overall, the high MAPE variance and mostly poor to moderate (AW) and poor (Fitbit) ICCs indicate a high risk of individual inaccuracy when monitoring HR from these wrist-worn SWs during wheelchair propulsion.

### Methodological and Future Considerations

Two main factors need to be addressed for better EE and HR estimation algorithms in wrist-worn SWs: (1) the sensor hardware and (2) the sensor software (estimation algorithm). The sensor hardware of current wrist-worn technology is not capable of reporting precise or consistent HR signals during activity, which was partly highlighted by the missing data in this study. With regard to further improving the estimation algorithms, an assessment is needed on the impact and relative importance of factors such as personal characteristics (sex, age, body mass, training status, etc) or more wheelchair users–specific aspects such as type and level of impairment. These additions may increase estimation accuracy and reduce variation within the highly heterogeneous wheelchair user group, even without hardware improvements.

Furthermore, investigation into appropriate cutoffs for acceptable accuracy of EE and HR provided by SWs is needed. The commonly used cutoffs (±3% in standardized and ±10% in free-living settings) are based on previous research assessing step count during walking [35-38] or pushes during wheelchair propulsion [39]. It may be plausible to establish higher cutoffs for EE or HR, especially when testing a heterogeneous wheelchair user group during an upper-body activity. However, a standardized low cutoff is essential to avoid differentiating acceptable accuracy between parameters in both controlled and free-living settings. Accurate estimates are also crucial for various populations, for example, to address an imbalance between energy intake and expenditure in athletes trying to regulate nutrition for performance purposes or in wheelchair users attempting to prevent or counteract obesity. Therefore, it is likely the ±10% error cutoff for free-living activities is too high.

Lastly, the effect of filtering done by the AW and Fitbit on the EE or HR data deserves mentioning. The AW removed many average HR values, which is likely attributed to a low number of data points. Comparatively, Fitbit reported data for all activities, although with lower accuracy. For now, we advise the use of HR belts for increased accuracy of the parameters investigated.

### Conclusion

Neither the wrist-worn AW nor Fitbit were sufficiently accurate for estimating EE or HR during wheelchair propulsion. The AW underestimated EE while the Fitbit overestimated the EE across all incline-stage combinations. The underestimation of the AW increased and the overestimation of the Fitbit decreased with higher intensities, suggesting that neither watch sufficiently adjusts for the change in intensity. Additionally, both SWs underestimated HR. High MAPEs were found for both SWs and parameters (ie, EE and HR), in addition to the poor relative agreement indicated by low ICCs. Furthermore, neither the wheelchair-specific algorithm for estimating EE nor its ability to differentiate between wheelchair users and people without a disability have been improved for the AW Series 4 as compared to the previously investigated AW Series 1. Overall, our findings suggest that caution is required when using SWs as a tool for training intensity regulation and energy balance or imbalance in wheelchair users.

## Appendix

Multimedia Appendix 1 Descriptive box plots comparing absolute values for criterion energy expenditure from Vyntus and hear rate from Polar to comparison devices Apple Watch and Fitbit. Data were split between wheelchair users and people without a disability, and individual-case differences were added in (shaded gray).

Multimedia Appendix 2 Bland-Altman plots visualizing absolute differences between criterion values and Apple or Fitbit values. The data were split between wheelchair users and people without disability and color coded for sex (male: blue circles, female: orange triangles). Furthermore, in accordance with Krouwer, criterion values were used on the x-axis rather than the average value of criterion and comparison device.

## Abbreviations

AW: Apple Watch
EE: energy expenditure
HR: heart rate
ICC: interclass correlation coefficient
MAPE: mean absolute percentage error
PAEE: physical activity energy expenditure
REE: resting energy expenditure
SCI: spinal cord injury
SW: smart watch

## Data Availability

The data sets generated during this study and corresponding analyses are available from the corresponding author on reasonable request. If used for further studies, the request includes a data application form and a data transfer agreement.
