# Supplementary material for: Accuracy of the Apple Watch Series 4 and Fitbit Versa for Assessing Energy Expenditure and Heart Rate of Wheelchair Users During Treadmill Wheelchair Propulsion: Cross-sectional Study
Source: JMIR Form Res. 2024 May 7;8:e52312. doi: 10.2196/52312 (PMC11109865; doi:10.2196/52312)

## Slide 1
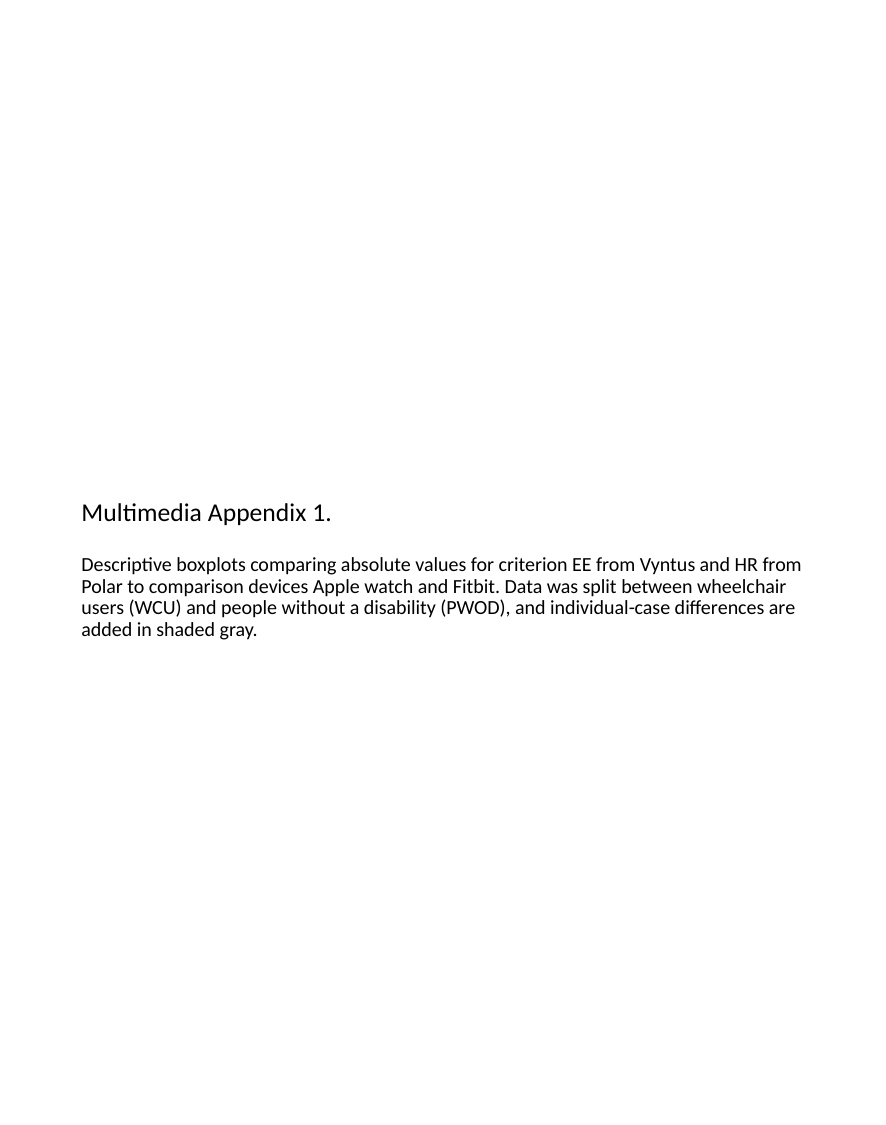

# Multimedia Appendix 1. Descriptive boxplots comparing absolute values for criterion EE from Vyntus and HR from Polar to comparison devices Apple watch and Fitbit. Data was split between wheelchair users (WCU) and people without a disability (PWOD), and individual-case differences are added in shaded gray.

## Slide 2
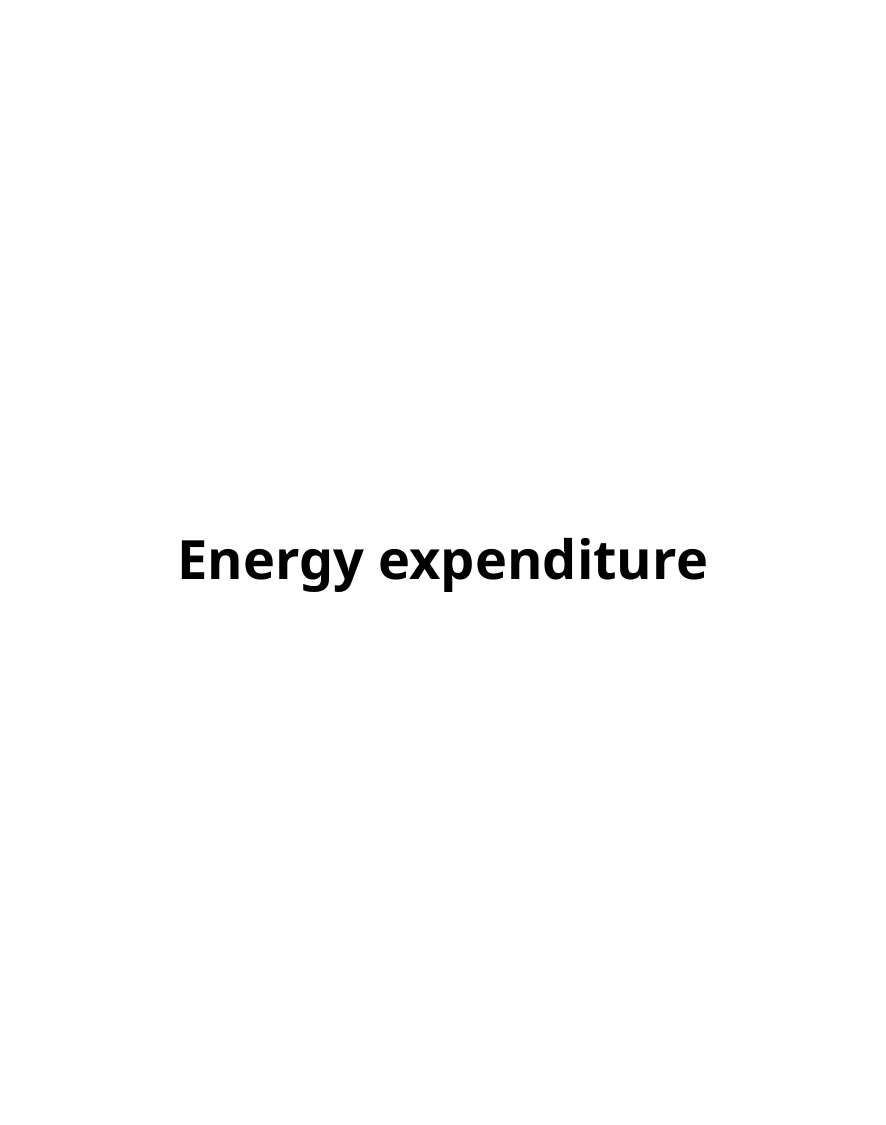

# Energy expenditure

## Slide 3
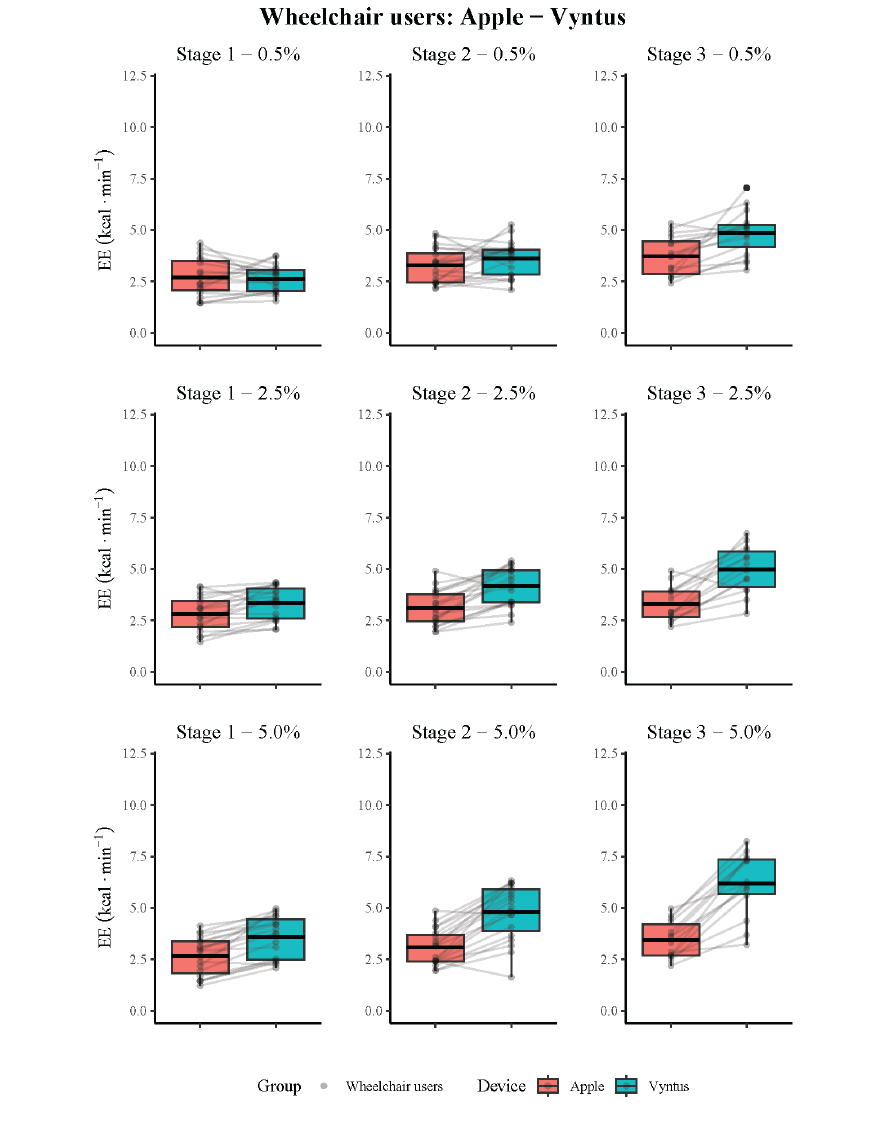

## Slide 4
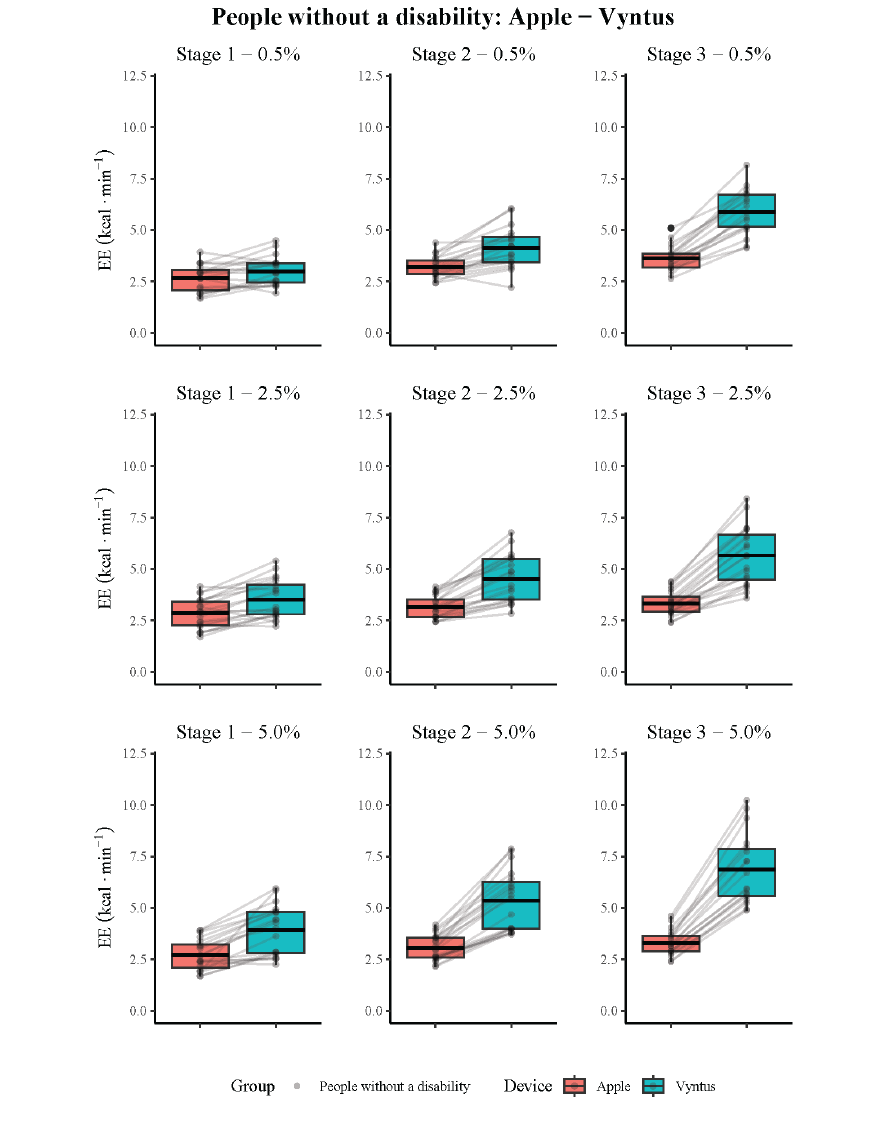

## Slide 5
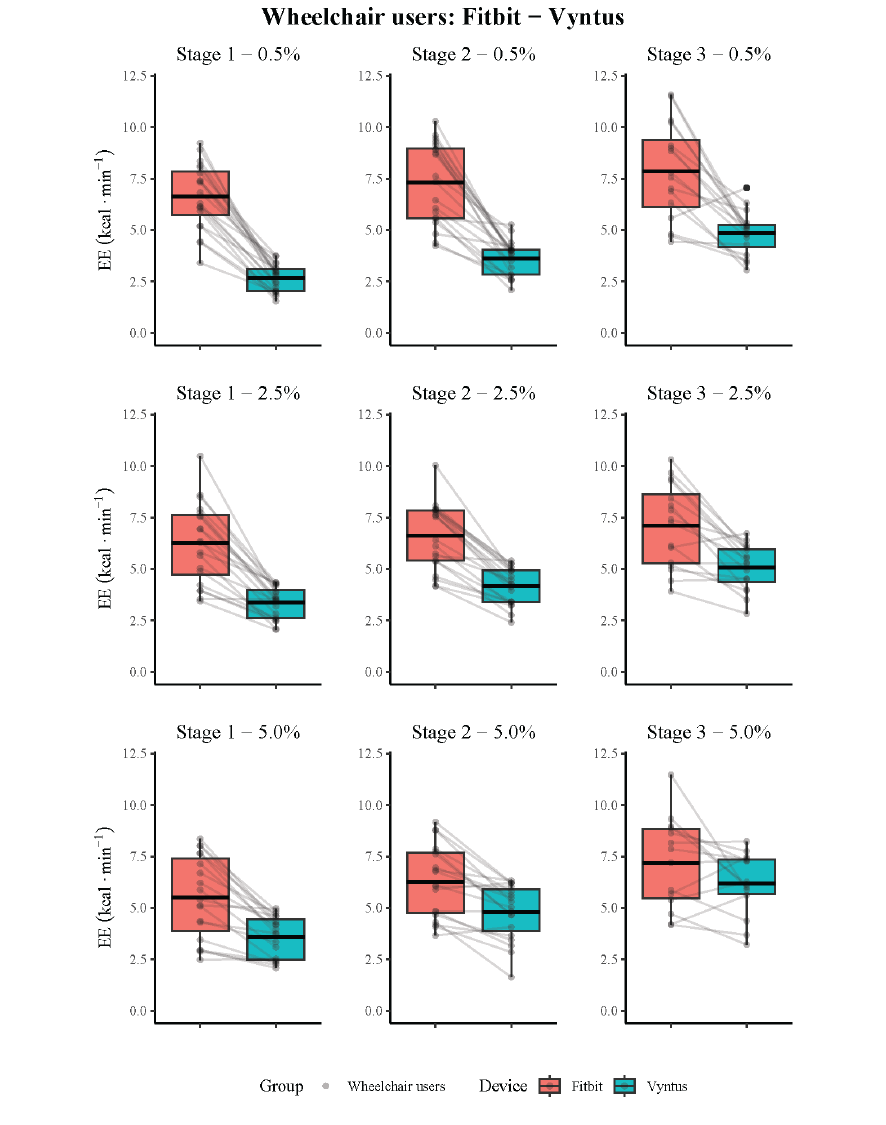

## Slide 6
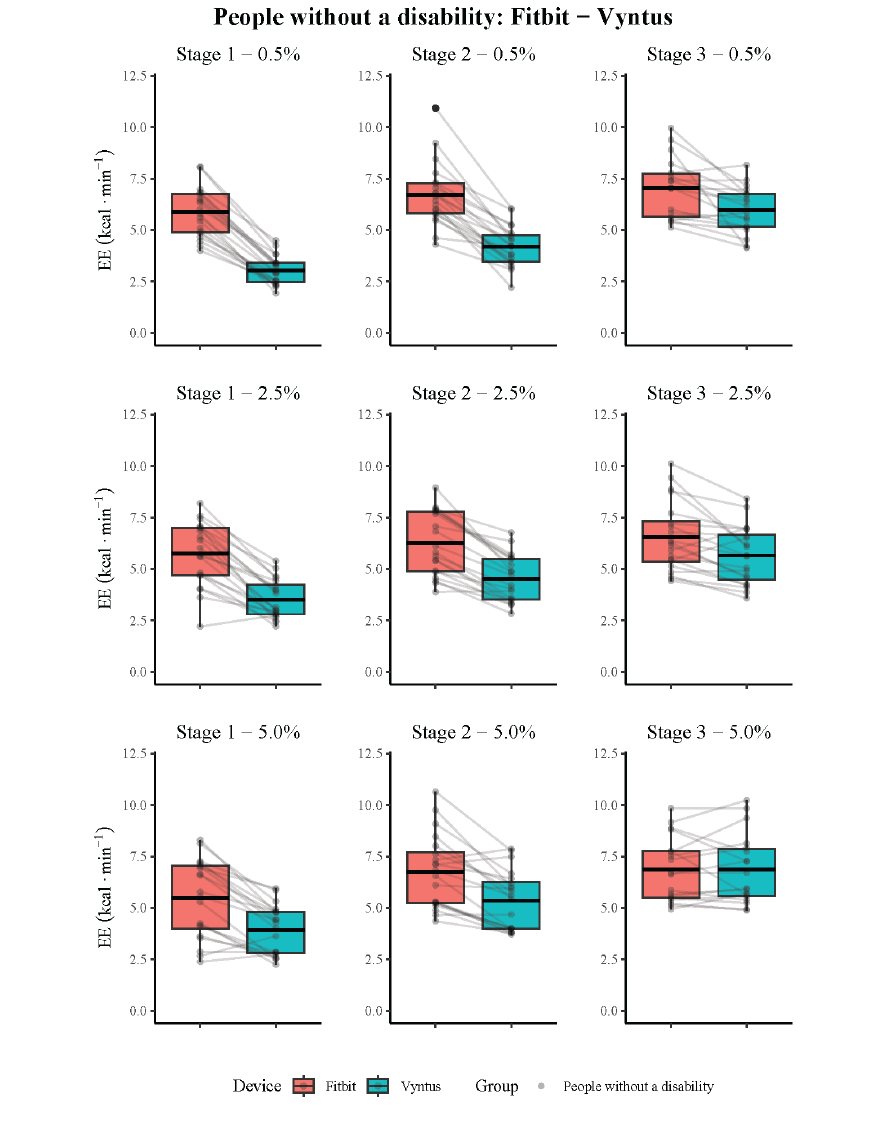

## Slide 7
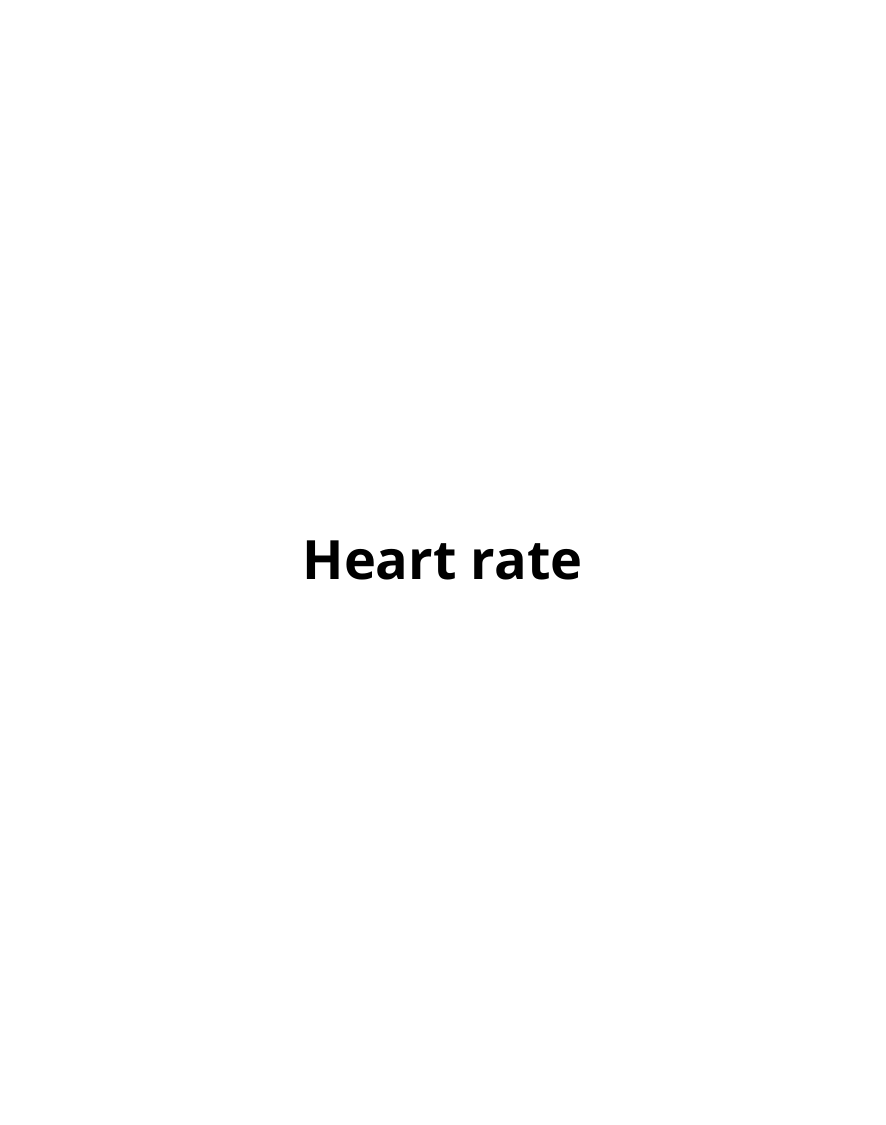

# Heart rate

## Slide 8
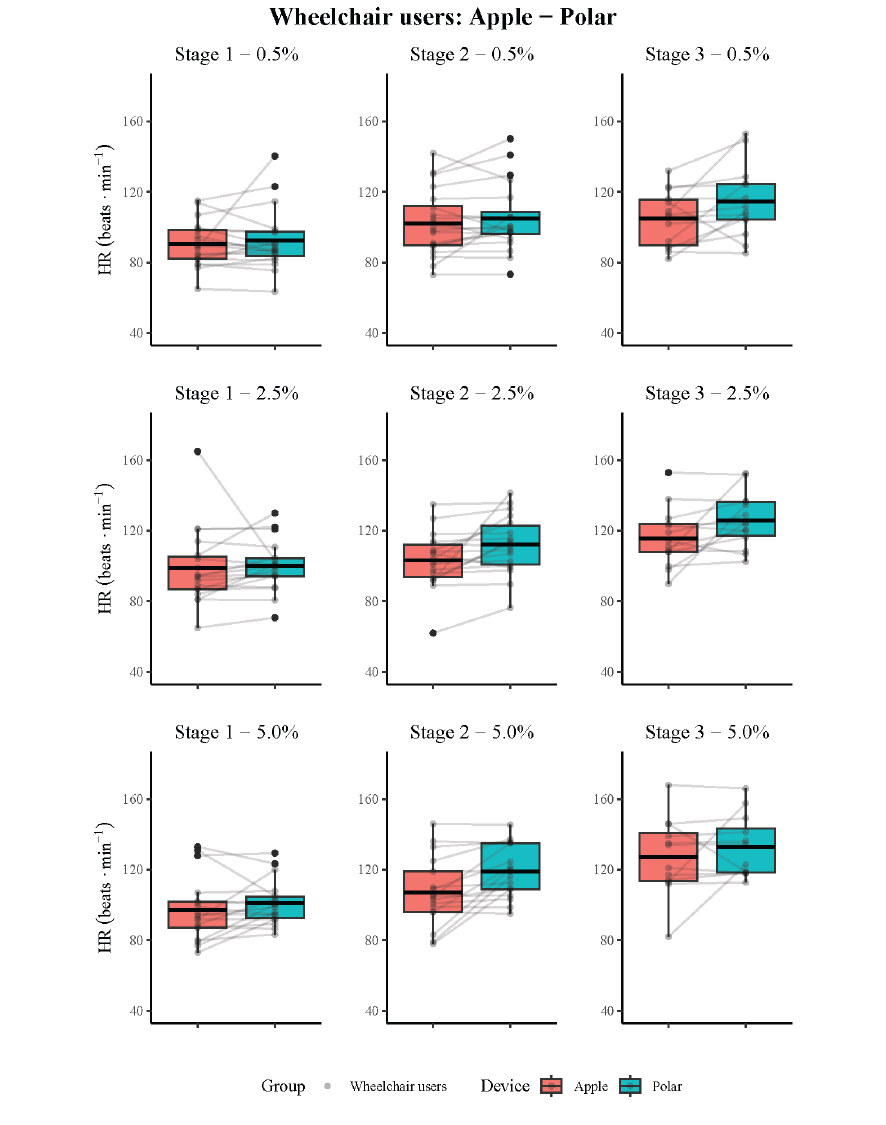

## Slide 9
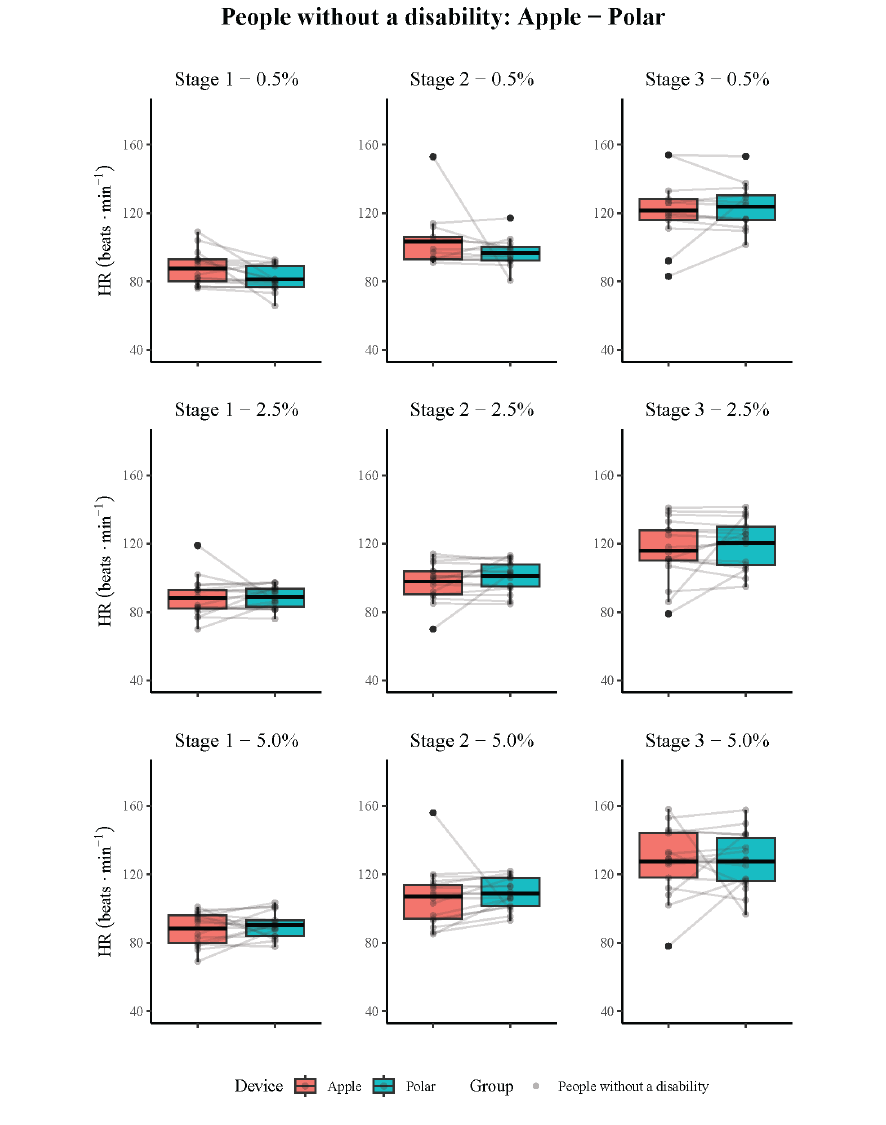

## Slide 10
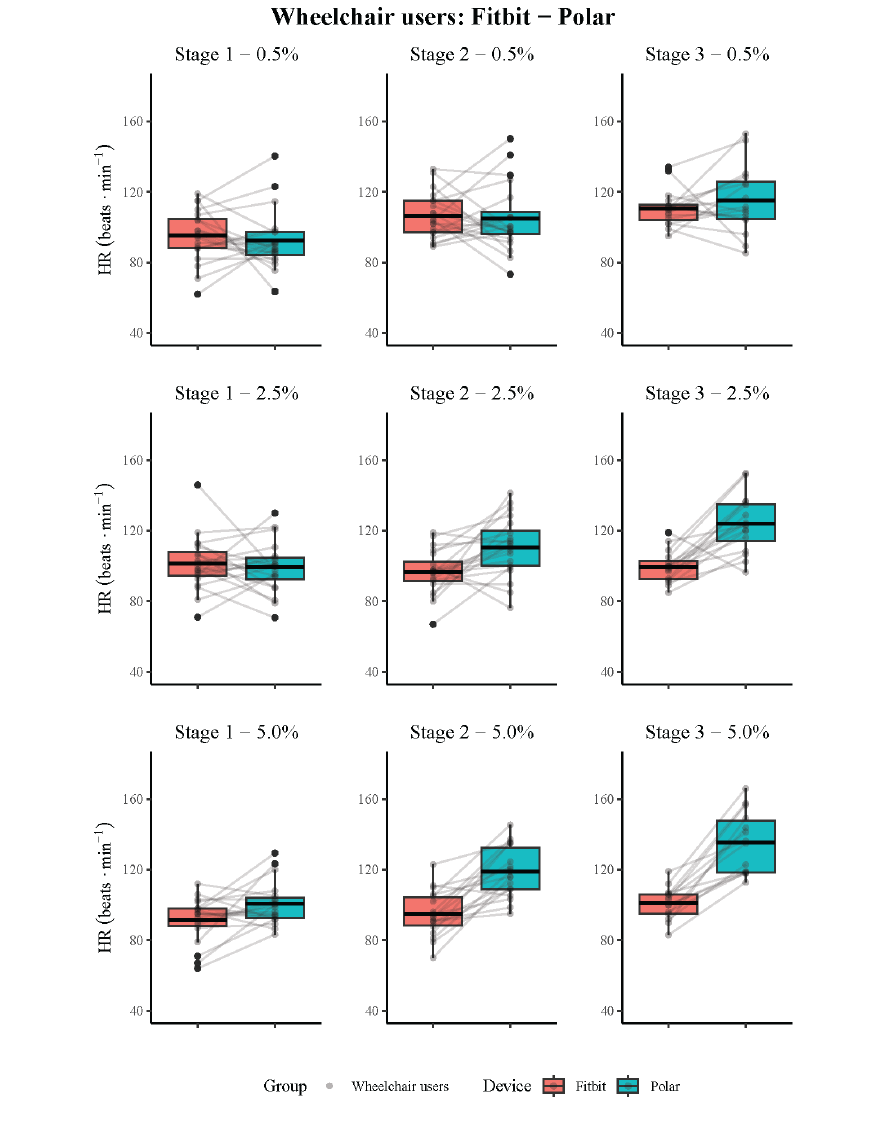

## Slide 11
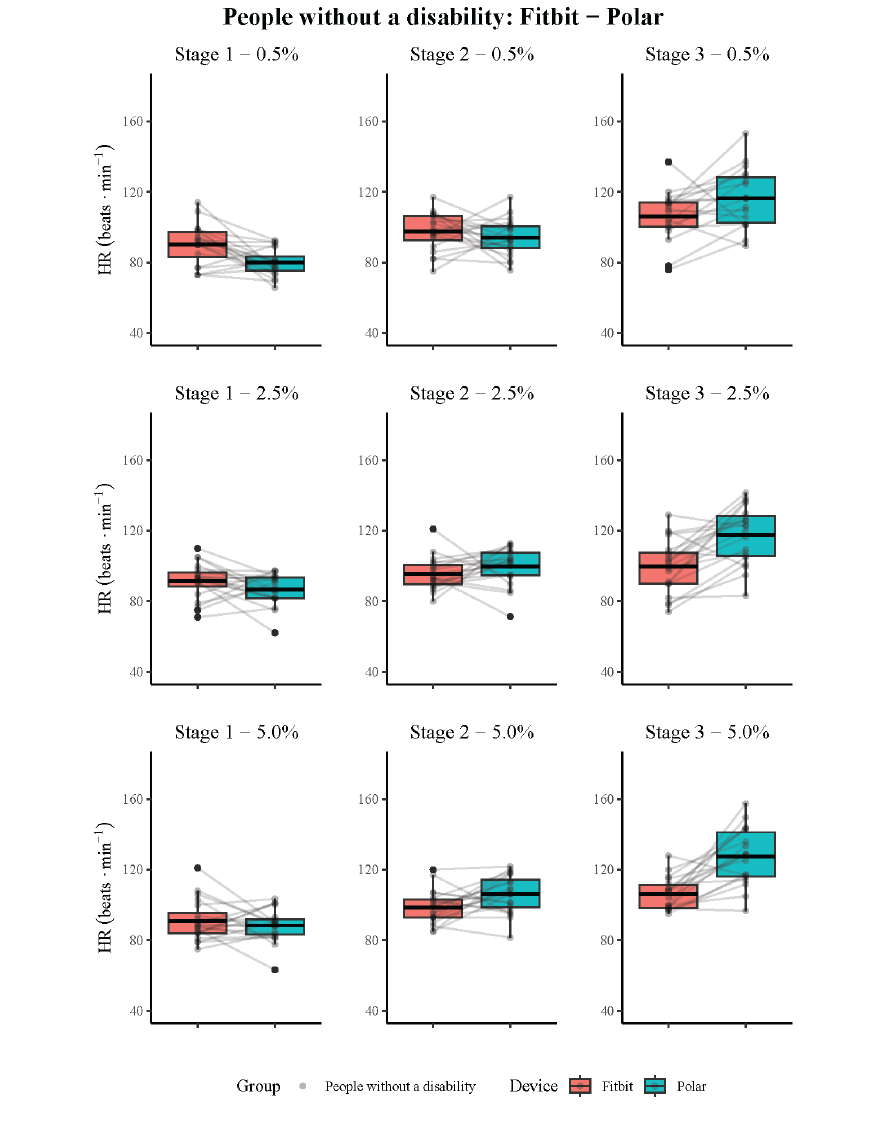

Supplement: Multimedia Appendix 1 [file formative_v8i1e52312_app1.pptx]
