# Supplementary material for: Accuracy of the Apple Watch Series 4 and Fitbit Versa for Assessing Energy Expenditure and Heart Rate of Wheelchair Users During Treadmill Wheelchair Propulsion: Cross-sectional Study
Source: JMIR Form Res. 2024 May 7;8:e52312. doi: 10.2196/52312 (PMC11109865; doi:10.2196/52312)

## Slide 1
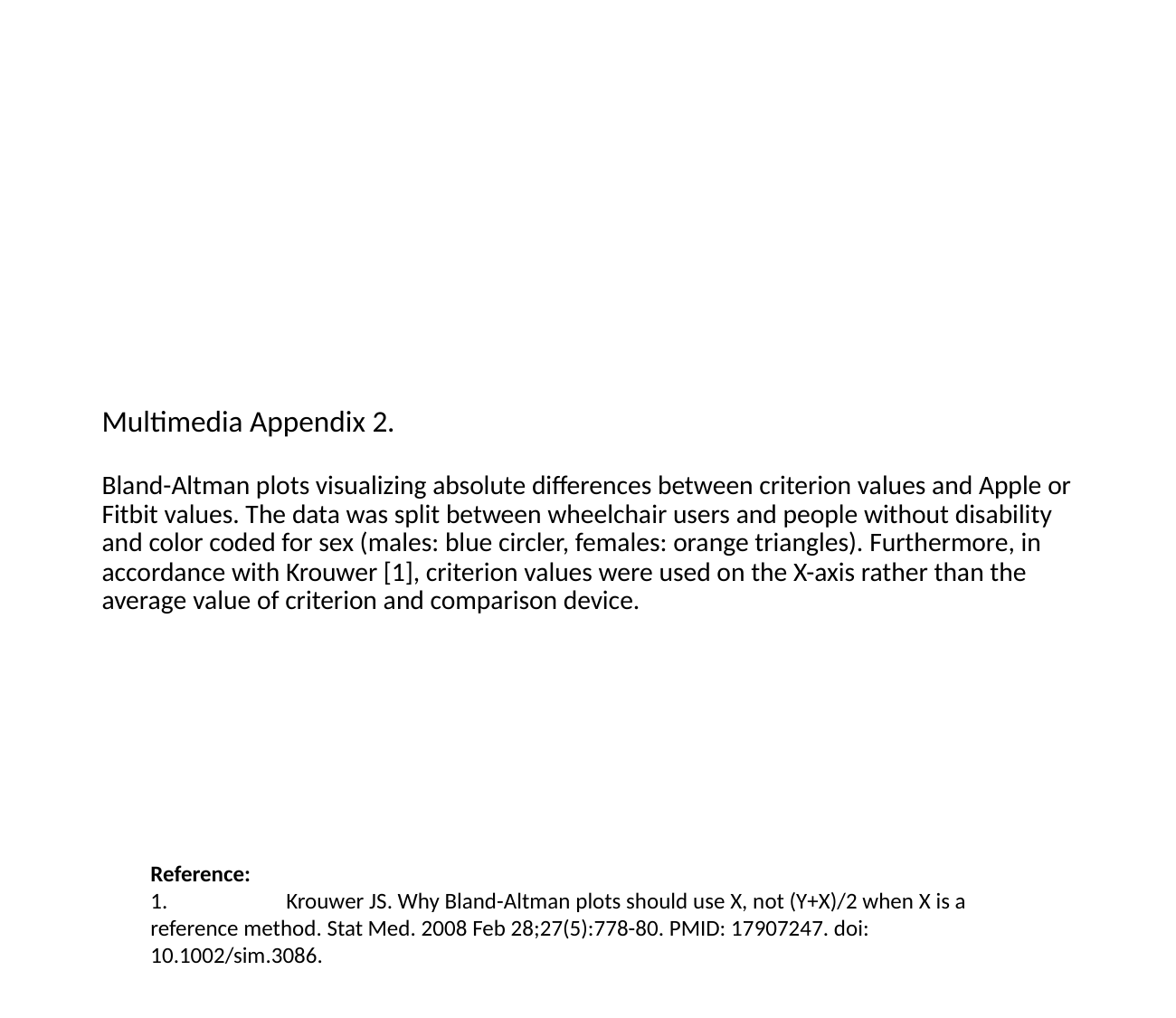

# Multimedia Appendix 2. Bland-Altman plots visualizing absolute differences between criterion values and Apple or Fitbit values. The data was split between wheelchair users and people without disability and color coded for sex (males: blue circler, females: orange triangles). Furthermore, in accordance with Krouwer [1], criterion values were used on the X-axis rather than the average value of criterion and comparison device.
Reference:
1.	Krouwer JS. Why Bland-Altman plots should use X, not (Y+X)/2 when X is a reference method. Stat Med. 2008 Feb 28;27(5):778-80. PMID: 17907247. doi: 10.1002/sim.3086.

## Slide 2
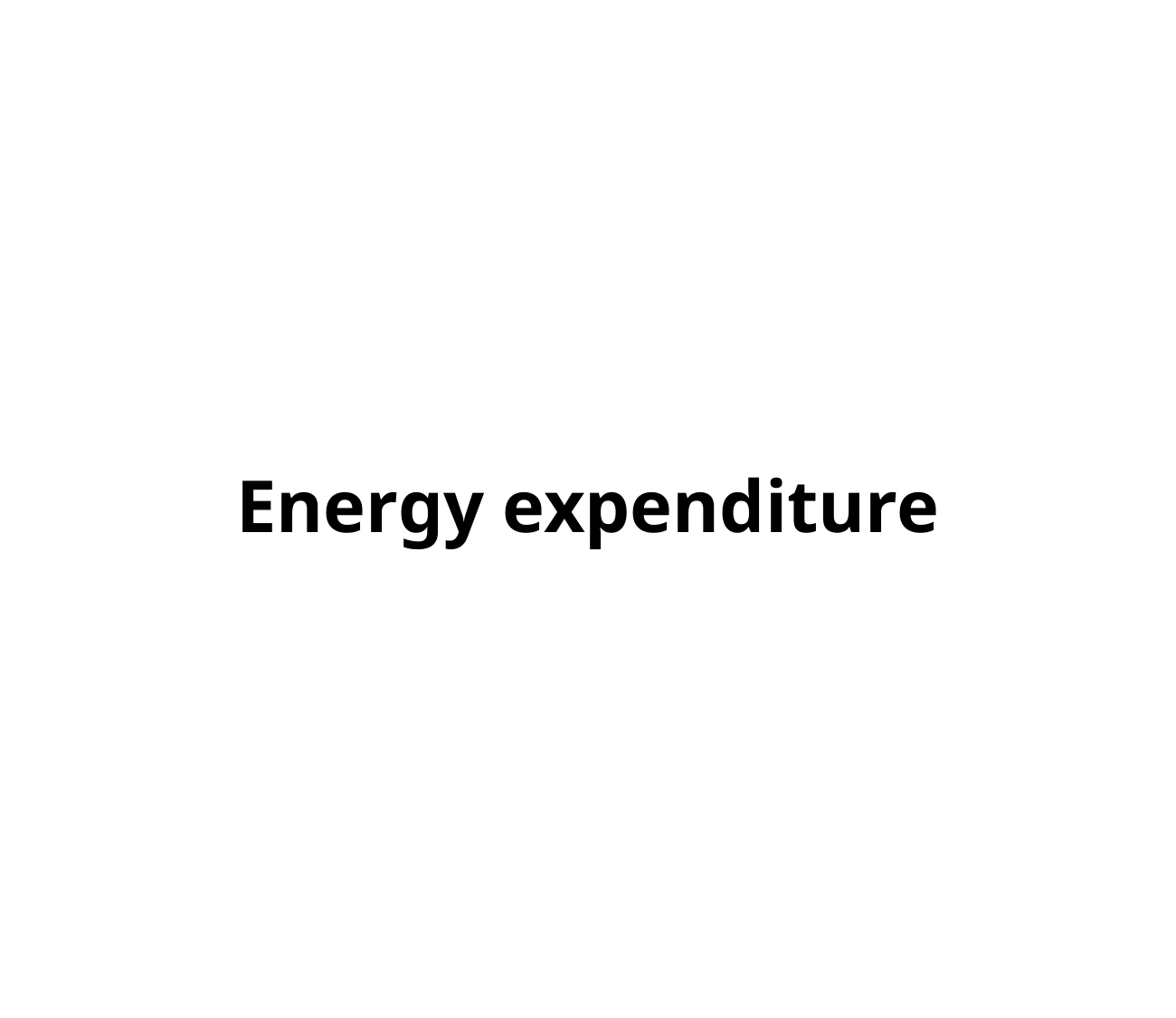

# Energy expenditure

## Slide 3
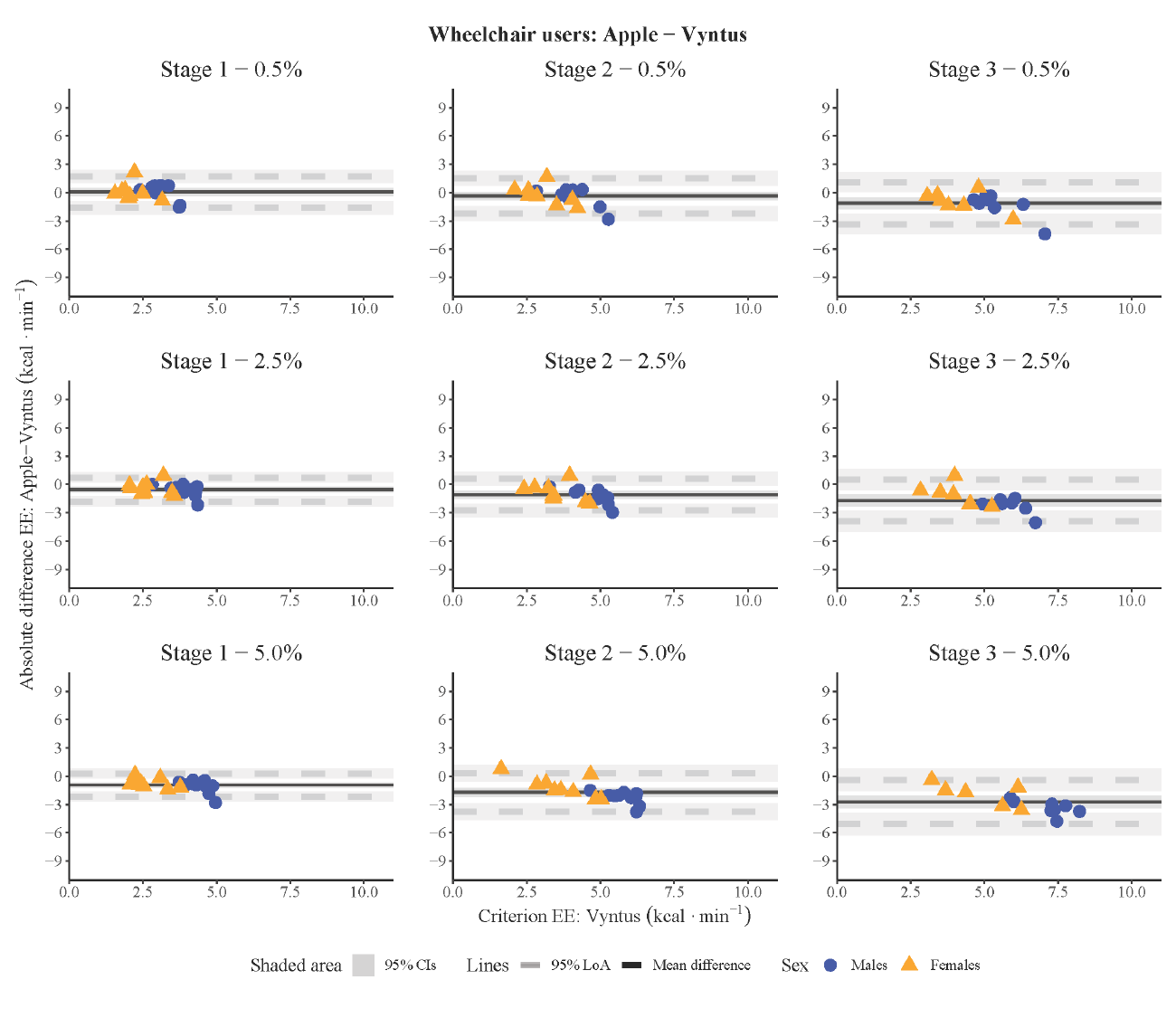

## Slide 4
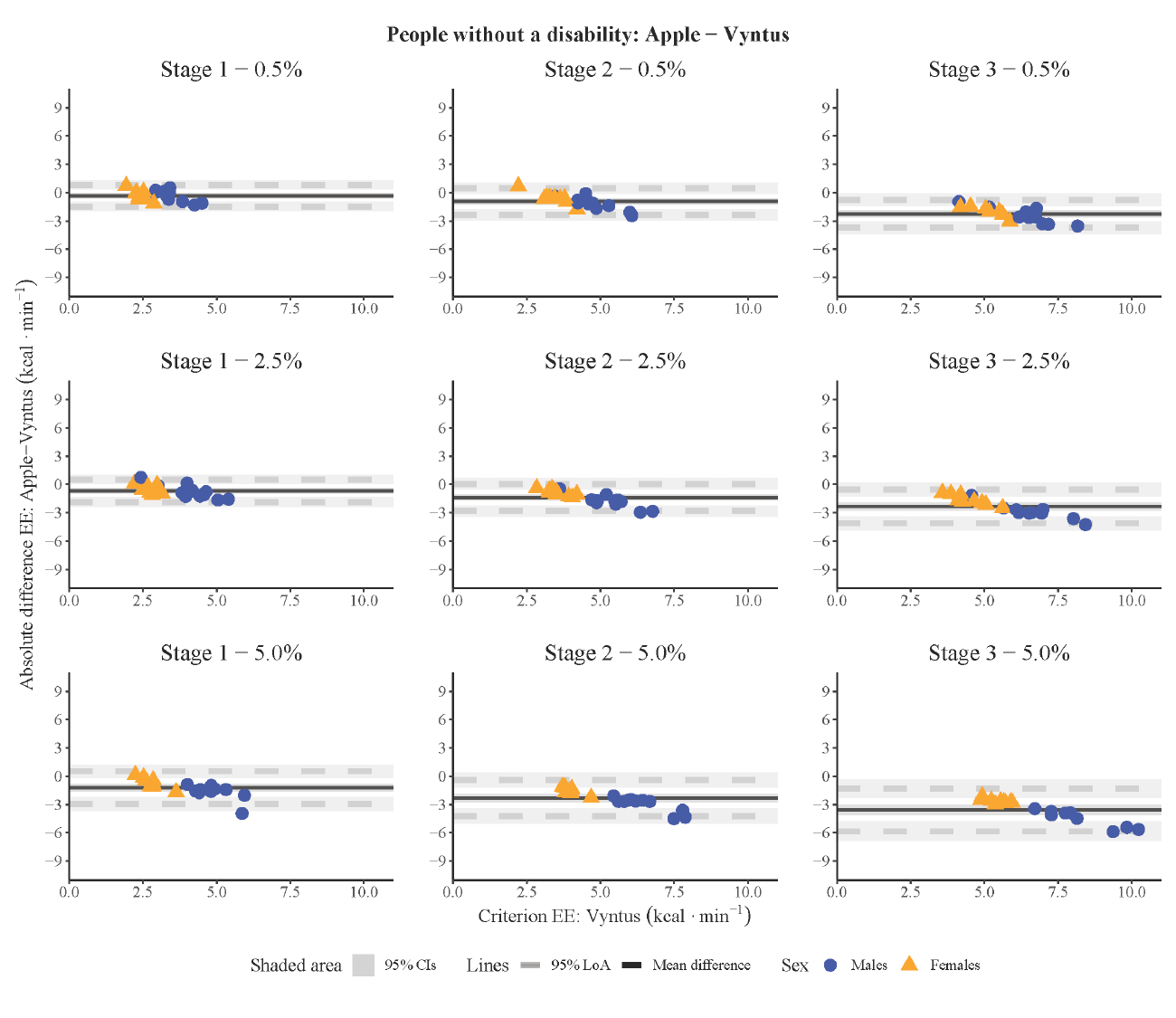

## Slide 5
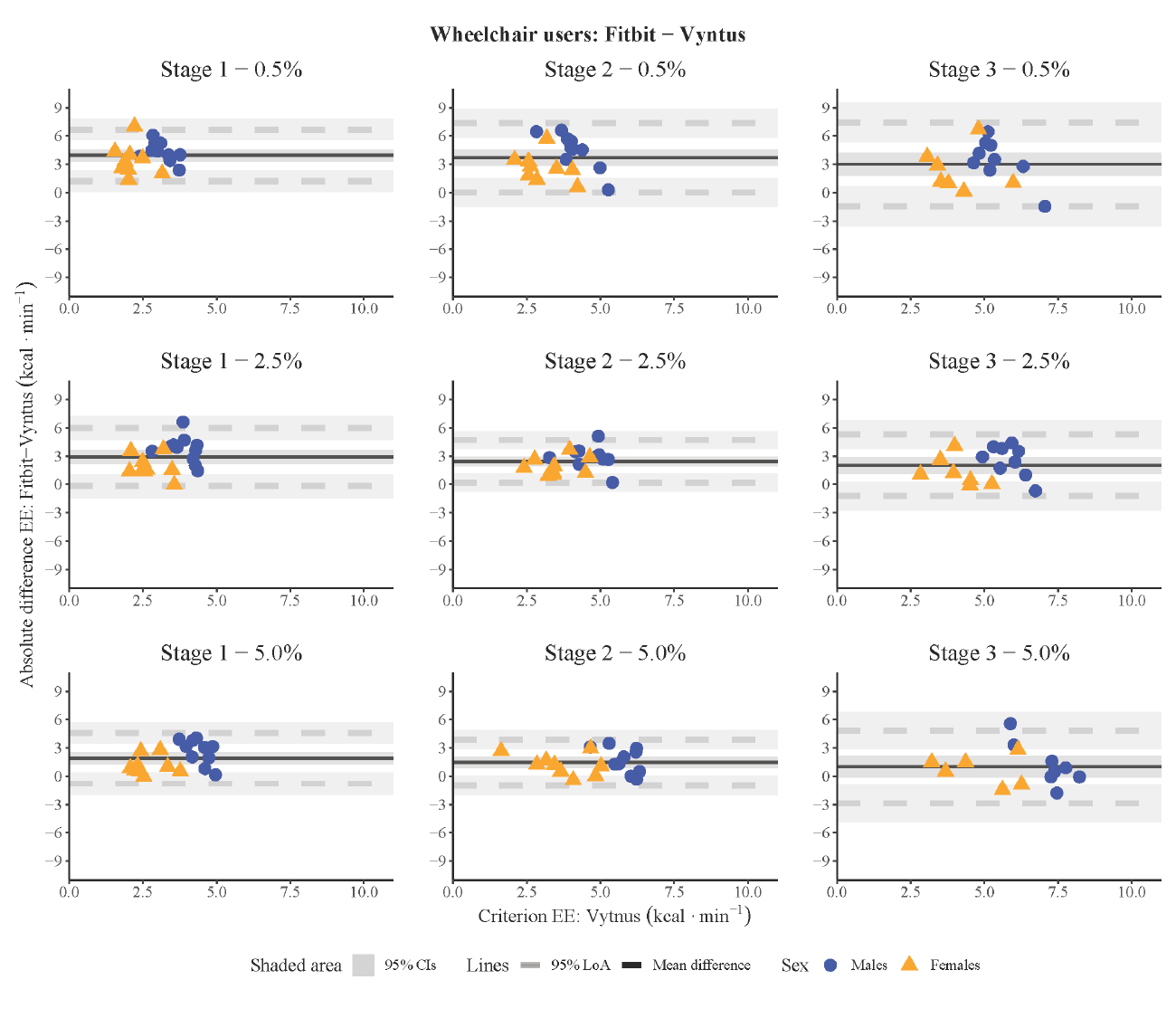

## Slide 6
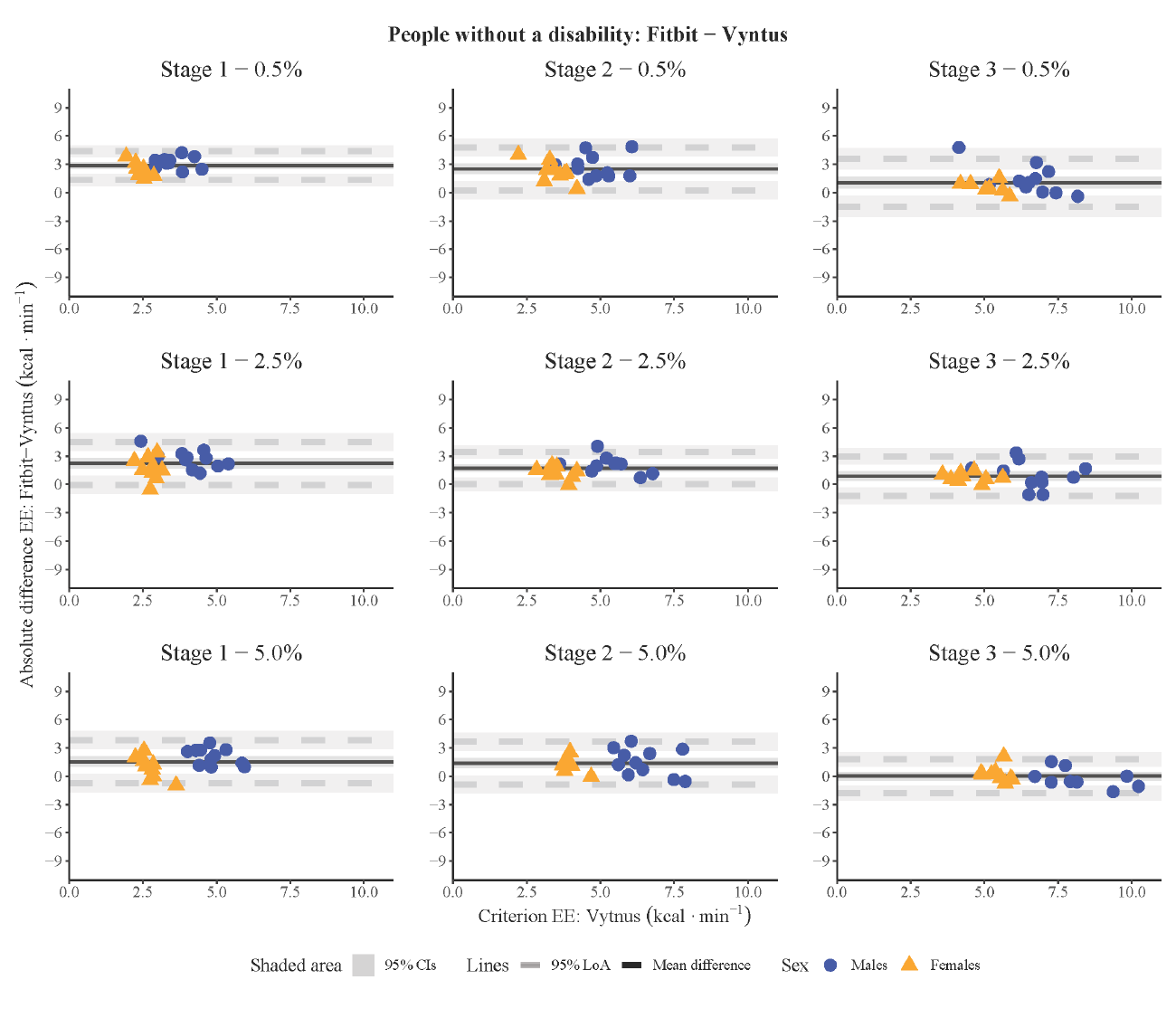

## Slide 7
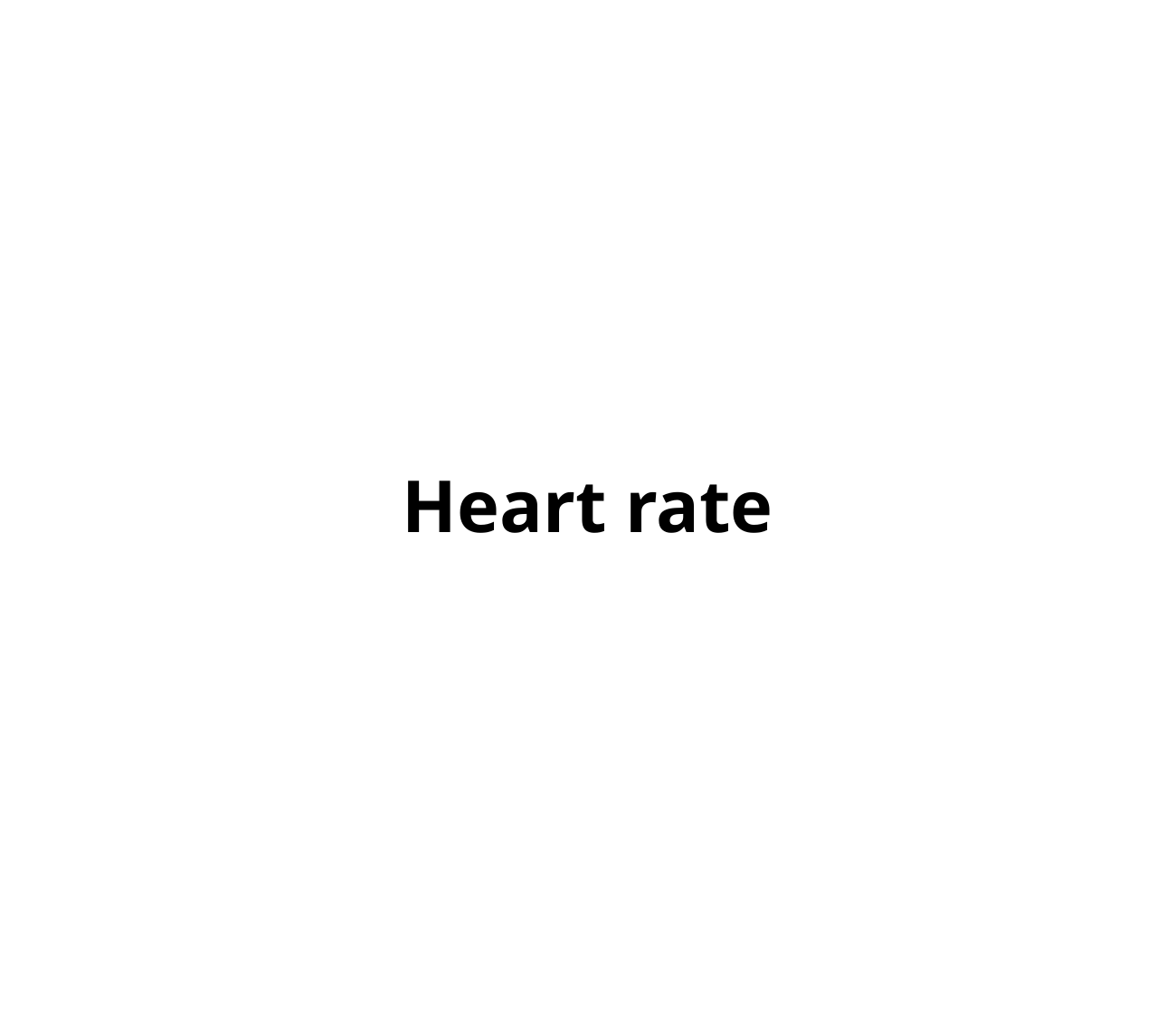

# Heart rate

## Slide 8
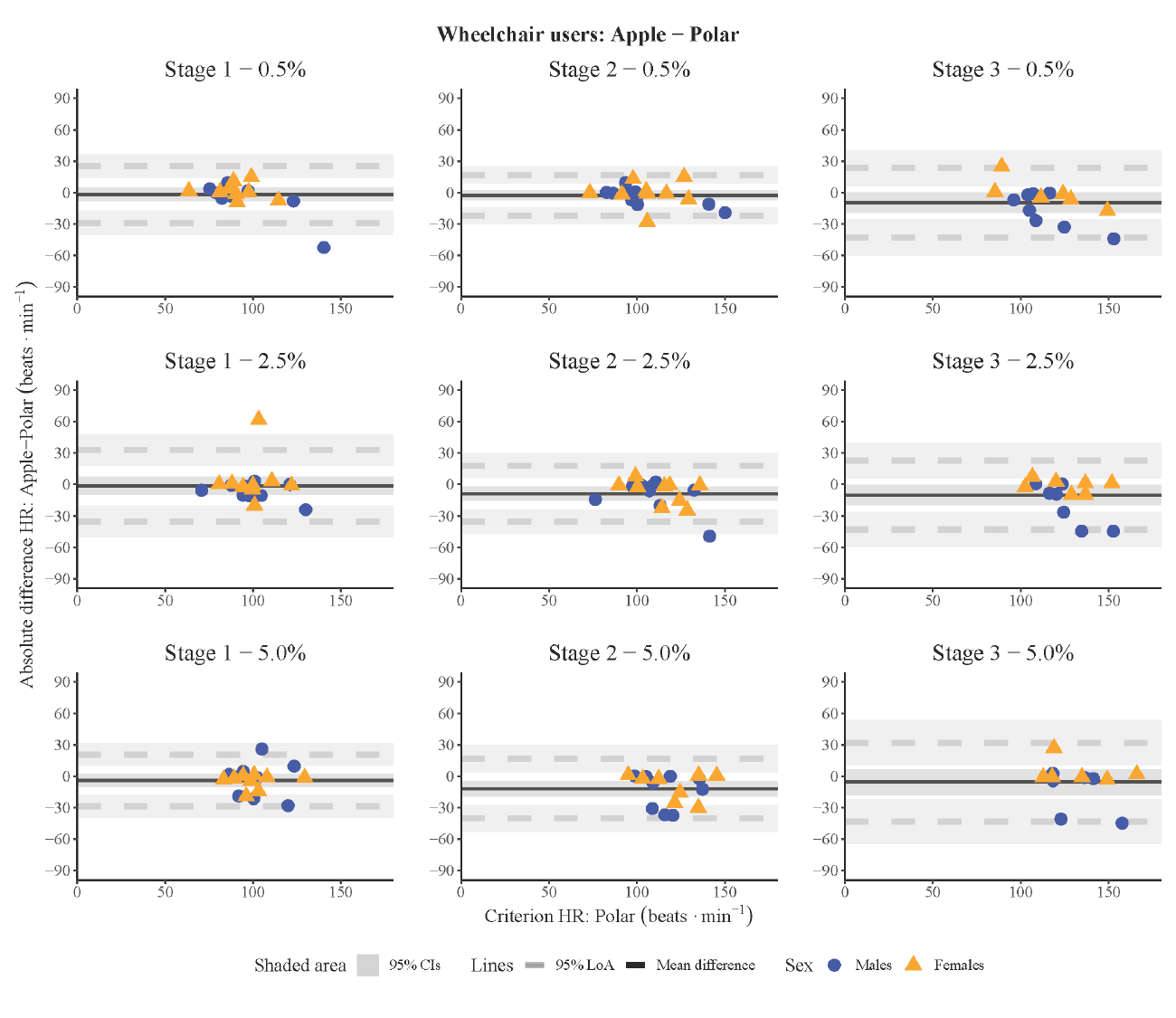

## Slide 9
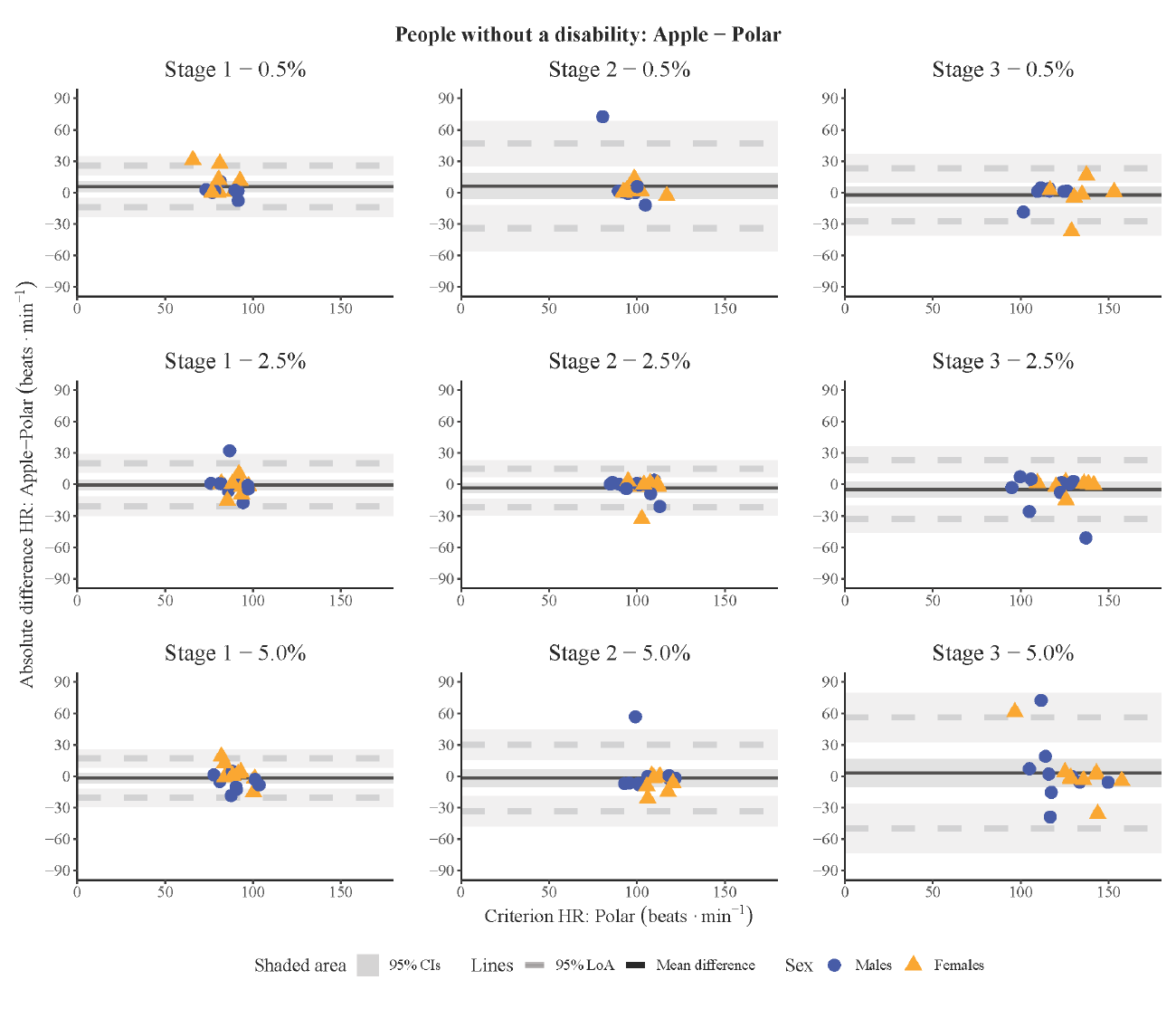

## Slide 10
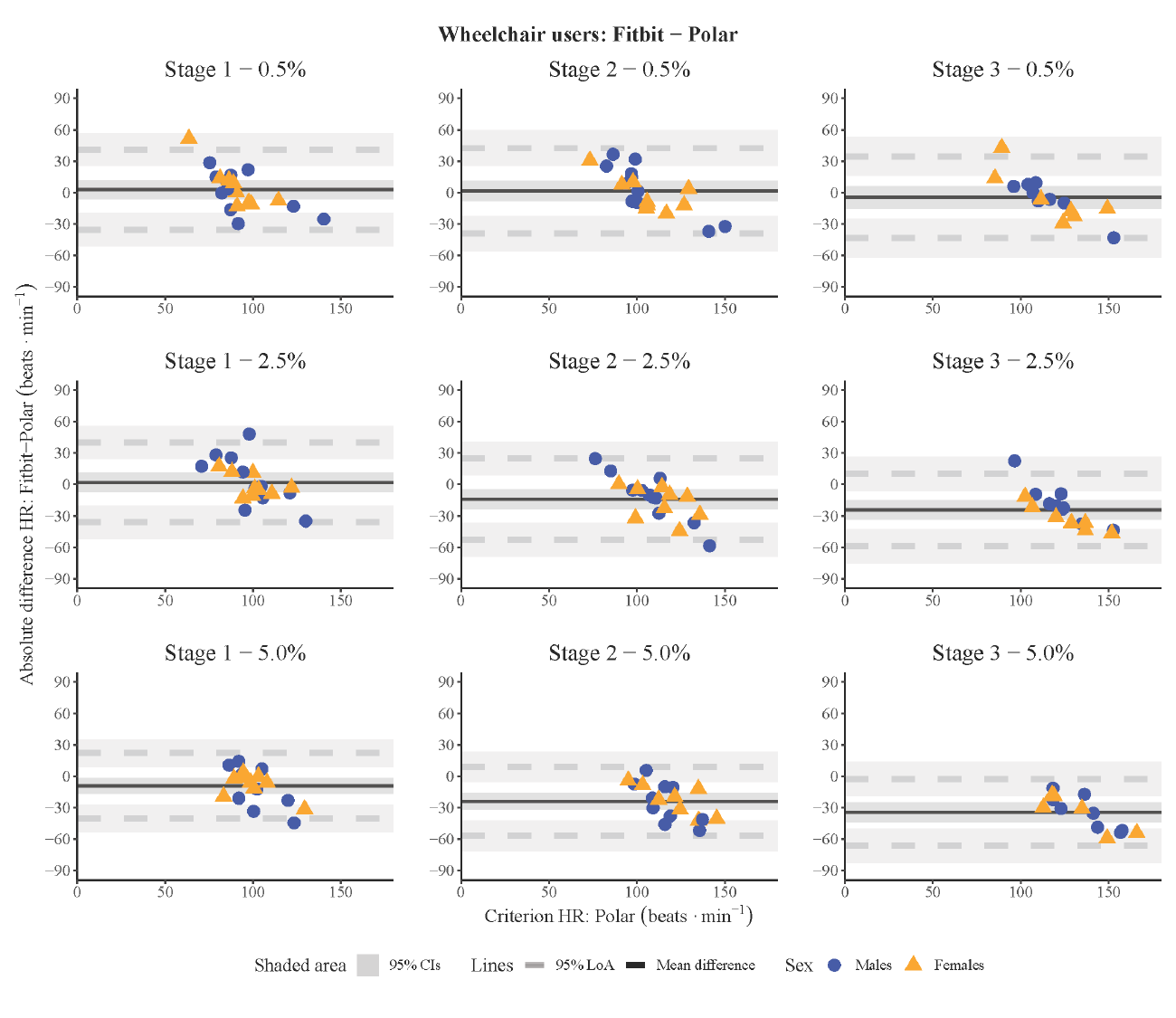

## Slide 11
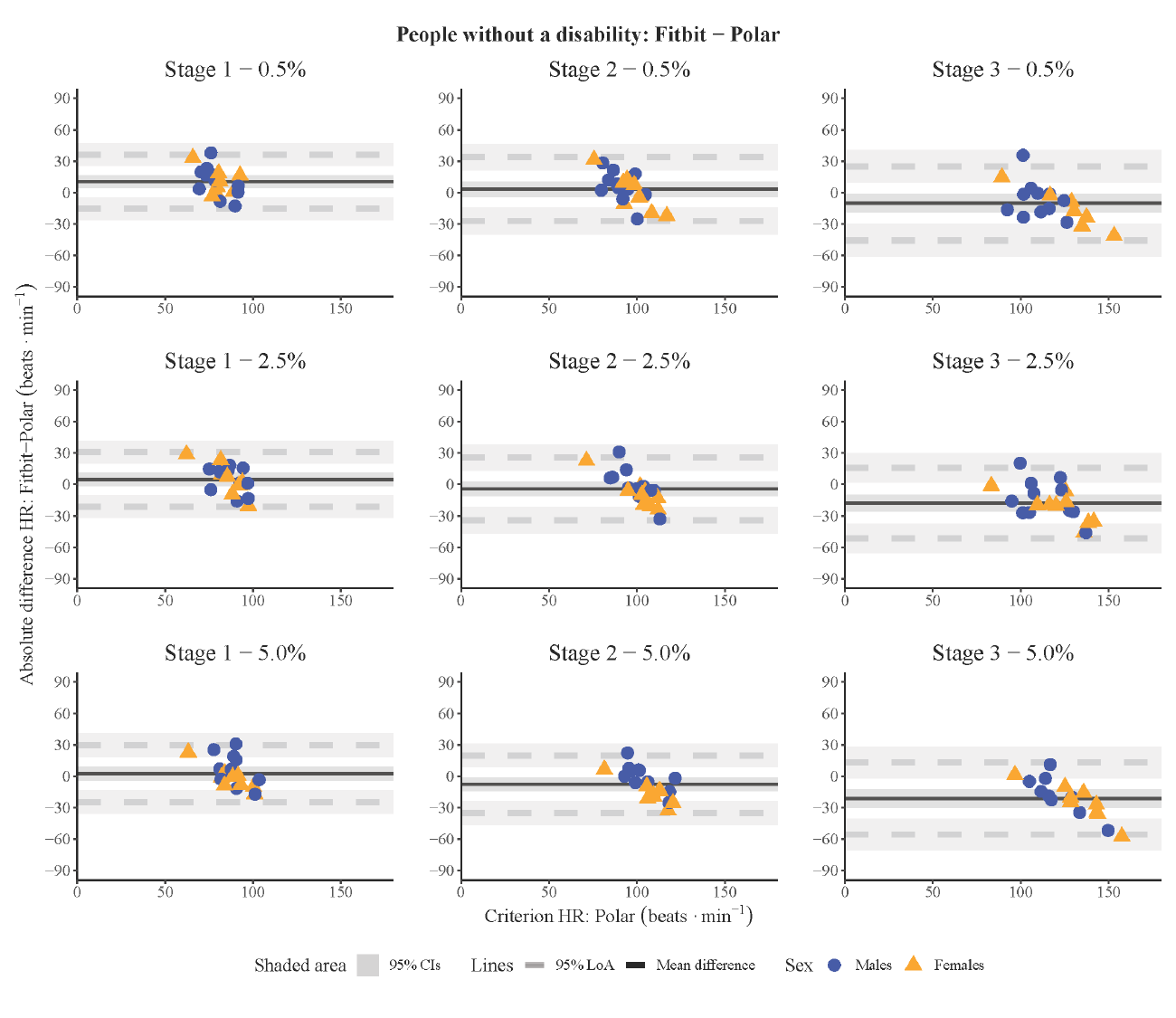

Supplement: Multimedia Appendix 2 [file formative_v8i1e52312_app2.pptx]
